# CD8+ T cell metabolic flexibility elicited by CD28-ARS2 axis-driven alternative splicing of *PKM* supports antitumor immunity

**DOI:** 10.1038/s41423-024-01124-2

**Published:** 2024-01-18

**Authors:** G. Aaron Holling, Colin A. Chavel, Anand P. Sharda, Mackenzie M. Lieberman, Caitlin M. James, Shivana M. Lightman, Jason H. Tong, Guanxi Qiao, Tiffany R. Emmons, Thejaswini Giridharan, Shengqi Hou, Andrew M. Intlekofer, Richard M. Higashi, Teresa W. M. Fan, Andrew N. Lane, Kevin H. Eng, Brahm H. Segal, Elizabeth A. Repasky, Kelvin P. Lee, Scott H. Olejniczak

**Affiliations:** 1 Department of Immunology, Roswell Park Comprehensive Cancer Center, Buffalo, NY 14263 USA; 2 Department of Medicine, Roswell Park Comprehensive Cancer Center, Buffalo, NY 14263 USA; 3 Human Oncology and Pathogenesis Program, Memorial Sloan Kettering Cancer Center, New York, NY 10065 USA; 4 Center for Environmental Systems Biochemistry, Department of Toxicology and Cancer Biology and Markey Cancer Center, Lexington, KY 40536 USA; 5 Department of Cancer Genetics and Genomics, Roswell Park Comprehensive Cancer Center, Buffalo, NY 14263 USA; 6 Present Address: University of Colorado Boulder, Boulder, CO 80309 USA; 7 Present Address: Dana Farber Cancer Institute, Boston, MA 02215 USA; 8 Present Address: Massachusetts Institute of Technology, Boston, MA 02139 USA; 9 Present Address: Melvin and Bren Simon Comprehensive Cancer Center, Indiana University School of Medicine, Indianapolis, IN 46202 USA

**Keywords:** Immunometabolism, mRNA splicing, CD8 T cells, ARS2, PKM2, Lymphocyte activation, Gene regulation in immune cells, Signal transduction

## Abstract

Metabolic flexibility has emerged as a critical determinant of CD8+ T-cell antitumor activity, yet the mechanisms driving the metabolic flexibility of T cells have not been determined. In this study, we investigated the influence of the nuclear cap-binding complex (CBC) adaptor protein ARS2 on mature T cells. In doing so, we discovered a novel signaling axis that endows activated CD8+ T cells with flexibility of glucose catabolism. ARS2 upregulation driven by CD28 signaling reinforced splicing factor recruitment to pre-mRNAs and affected approximately one-third of T-cell activation-induced alternative splicing events. Among these effects, the CD28-ARS2 axis suppressed the expression of the M1 isoform of pyruvate kinase in favor of PKM2, a key determinant of CD8+ T-cell glucose utilization, interferon gamma production, and antitumor effector function. Importantly, *PKM* alternative splicing occurred independently of CD28-driven PI3K pathway activation, revealing a novel means by which costimulation reprograms glucose metabolism in CD8+ T cells.

## Introduction

Antitumor responses of CD8+ T cells rely on coordinated regulation of their transcriptional output, driving their growth, proliferation, and acquisition of effector properties [1]. These bioenergetically demanding processes are enabled by the multiphasic metabolic reprogramming that occurs following naïve T-cell activation [2]. Within 24 h of activation, glycolysis is rapidly induced through T-cell receptor (TCR)-driven phosphorylation of pyruvate dehydrogenase kinase 1 (PDHK1) [3] and subsequent CD28/phosphoinositide 3-kinase (PI3K)-dependent upregulation of glucose transporters [4]. However, early induction of glycolytic flux is insufficient to support CD8+ T-cell effector functions [5]. Rather, a continued increase in glycolytic flux supports posttranscriptional upregulation of the key effector cytokines interferon gamma (IFNγ), tumor necrosis factor alpha (TNFα), and interleukin-2 (IL-2) by limiting the binding of the glycolytic enzymes lactate dehydrogenase (LDH) and/or glyceraldehyde-3-phosphate dehydrogenase (GAPDH) to AU-rich elements in the 3′ untranslated regions of coding mRNAs [3, 5].

Several recent studies have suggested that another enzyme involved in glycolysis, pyruvate kinase M2 (PKM2), also has a “moonlighting” function in T cells; PKM2 translocates to the nucleus in activated CD4+ T cells, where it regulates transcription and thus affects Th17 differentiation [6–8]. A direct transcriptional regulatory function of PKM2 has also been reported in tumor cells, although this nonmetabolic function remains controversial [9]. The metabolic function of PKM2 is central to the Warburg effect in tumor cells and was also recently shown to be important for natural killer (NK) cell function [10]. In tumor cells, PKM2 expression is increased by mutually exclusive alternative splicing to favor the inclusion of exon 10 over exon 9 due to oncogene-driven changes in the expression of the splicing factors hnRNPA1/A2 and PTBP1 that suppress exon 9 inclusion, and in the expression of SRSF3, which directs the inclusion of exon 10. Functionally, the PKM1 and PKM2 proteins differ primarily in the rate at which they convert phosphoenolpyruvate (PEP) to pyruvate in cells, with slower conversion via PKM2 leading to the accumulation of glycolytic intermediates used for the anabolic synthesis of macromolecules. The disparity in pyruvate kinase activity between PKM1 and PKM2 is not due to inherent differences in their enzymatic activity but rather due to the regulation of PKM2 tetramer formation by a number of allosteric activators or inhibitors, including PEP, fructose-1,6-bisphosphate, alanine, ATP, serine, and SAICAR, or by direct posttranslational modification of PKM2 [9]. Despite evidence that PKM2 expression is strongly induced in activated lymphocytes [6–8, 10], whether similar alternative splicing mechanisms regulate PKM2 induction in activated T cells remains unclear, as does the consequence of PKM2 upregulation in CD8+ T cells.

Alternative splicing relies not only on splicing factors but also on cotranscriptional 5′ modifications of pre-mRNAs with a 7-methylguanosine (7-mG) cap and subsequent recruitment of the nuclear cap-binding complex (CBC; composed of CBP80 and CBP20), which controls virtually all aspects of RNA processing, including splicing, 3′ end processing, nuclear export, RNA degradation, and translation [11]. The CBC adaptor protein arsenic resistance protein 2 (ARS2) serves as a scaffold between the CBC and RNA processing factors involved in RNA 3′ end processing and nuclear export or RNA degradation [12–14]. ARS2 is an essential protein that plays context-specific roles in mammalian physiology, including roles in the maintenance of neural and hematopoietic stem cell identity [15, 16], retinal progenitor cell proliferation [17], and thymocyte survival [16]. Unlike that of other CBC components, the expression of ARS2 changes in response to extracellular stimuli [18–20], suggesting that regulation of ARS2 expression may be a means for cells to coordinate signaling cues with transcriptional output.

In the present study, we investigated the role of ARS2 in CD8+ T cells. We found that ARS2 was induced in the tumor microenvironment and tumor-draining lymph nodes by T-cell activation in a CD28 costimulation-dependent manner and was necessary for antigen-specific control of tumor growth in mouse models. Rather than having significant effects on gene expression, which is largely dictated by T-cell receptor (TCR) signaling [21–23], CD28-driven ARS2 upregulation primarily affects alternative splicing. Among the ARS2 splicing targets, we identified the mRNA encoding pyruvate kinase (*Pkm*) as a critical ARS2 effector and showed that CBC-bound ARS2 orchestrates the association of splicing factors with *Pkm* pre-mRNA to suppress exon 9 inclusion and thereby favor the expression of the M2 isoform of pyruvate kinase (PKM2) in activated T cells. ARS2-directed *Pkm* splicing occurs days after upregulation of glucose transporters in activated T cells and limits the incorporation of glucose carbons into metabolites derived from pyruvate, including alanine, lactate, and proximal tricarboxylic acid (TCA) cycle metabolites. Inhibition of lymphocyte-specific protein tyrosine kinase (LCK) signaling or mutation of the membrane-distal CD28 PYAP intracellular signaling domain, which is responsible for LCK activation, blocked ARS2 upregulation and *Pkm* splicing, while rescue of ARS2 or PKM2 expression in CD28 PYAP domain-mutant T cells restored effector cytokine production and flexibility in glucose utilization. Together, these data reveal a novel CD28-ARS2-PKM2 axis involved in reprogramming glucose metabolism and increasing effector cytokine production to support CD8+ T-cell-mediated antitumor immunity.

## Results

### The ARS2-bound CBC (CBCA) supports CD8+ T-cell-mediated antitumor immunity

**Fig. 1:**
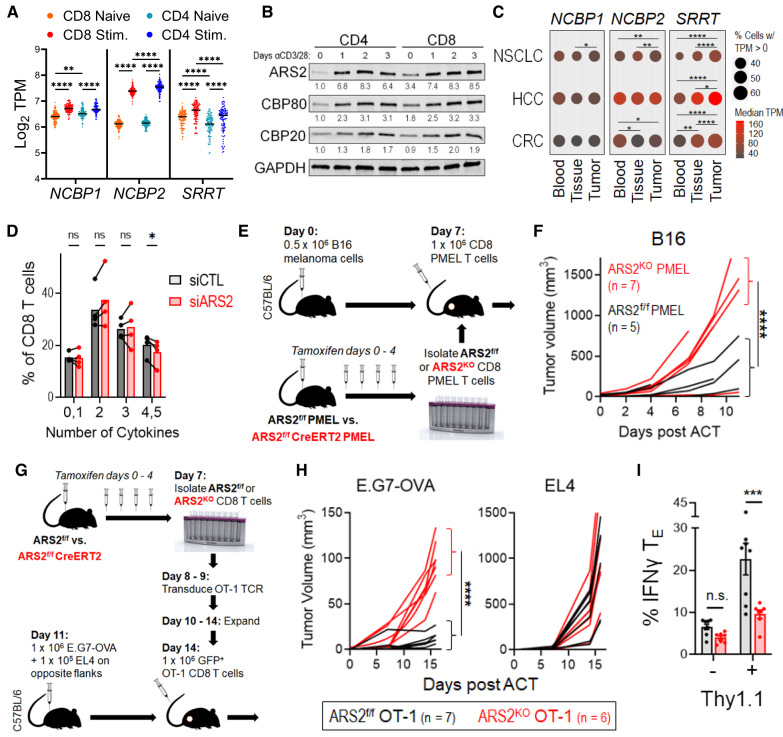
ARS2 supports CD8+ T-cell-mediated antitumor immunity **A** Violin plot showing the mRNA expression of the CBCA components CBP80 (*NCBP1*), CBP20 (*NCBP2*), and ARS2 (*SRRT*) in naïve compared with stimulated human CD4^+^ and CD8^+^ T cells; the data are from the DICE database (https://dice-database.org/). **B** Western blots showing the expression of CBCA components in separately isolated CD4^+^ or CD8^+^ human T cells stimulated with αCD3/αCD28 microbeads for the indicated number of days. The blots are representative of two healthy human donors. **C** Frequency (circle size) and magnitude (color) of CBCA component mRNA expression in single CD8+ T cells isolated from blood, adjacent normal tissue, or tumor tissue of patients with non-small cell lung cancer (NSCLC, data from GSE99254), hepatocellular carcinoma (HCC, data from GSE98638), or colorectal carcinoma (CRC, data from GSE108989). **D** Frequency of control compared with ARS2 siRNA-transfected healthy human donor CD8+ T cells expressing the indicated number of cytokines 3 days post transfection and stimulation with αCD3/αCD28 beads. Prior to intracellular staining for the cytokines shown in Supplementary Fig. 1J, T cells were restimulated for 4 h with PMA + ionomycin in the presence of brefeldin A. The bars indicate the means of 4 healthy human donors, and the connected points represent individual donors. **E** Schematic depicting the PMEL adoptive cell therapy (ACT) model used to compare antigen-specific antitumor immunity between ARS2^f/f^ and ARS2^KO^ CD8+ T cells. **F** Growth of subcutaneous B16 tumors following ACT with ARS2^f/f^ (black line) or ARS2^KO^ (red line) CD8+ T cells isolated from PMEL TCR transgenic mice. **G** Schematic depicting the ACT model used to compare antigen-specific antitumor immunity between ARS2^f/f^ and ARS2^KO^ CD8+ T cells following transduction with the OT-1 TCR transgene. **H** Growth of subcutaneous tumors following ACT with ARS2^f/f^ (black line) or ARS2^KO^ (red line) OT-1 TCR-transduced T cells. Isogenic E.G7-OVA OT-1 target and EL4 control tumor cells were implanted into opposite flanks. **I** IFNγ expression in E.G7-OVA tumor-infiltrating OT-1-transduced (Thy1.1^+^) and bystander (Thy1.1^−^) ARS2^f/f^ (gray bars) or ARS2^KO^ (red bars) T_E_ cells. The bars indicate the means ± SDs, and the dots represent biological replicates. The lines in (**F**) and (**H**) represent individual tumors. Differences between groups were determined by ANOVA (**A**, **C**, **D**, **I**) or mixed effects analysis (**F**, **H**). ns = not significant, *p < 0.05, **p < 0.01, ***p < 0.001, ****p < 0.0001

Unlike in other cell types where ARS2 is the only regulated CBCA component [12, 18, 20], T-cell activation with αCD3/αCD28 microbeads + recombinant interleukin-2 (rIL-2) resulted in upregulation of ARS2 (*Srrt*), CBP80 (*Ncbp1*), and CBP20 (*Ncbp2*) mRNA and protein expression (Supplementary Fig. 1A, B). Activation-induced CBCA upregulation occurred in both CD4+ and CD8+ T cells, with human and mouse CD8+ T cells exhibiting greater ARS2 expression than CD4+ T cells (Fig. 1A, B and Supplementary Fig. 1C). Transcriptional upregulation of ARS2 was independent of biological sex (Supplementary Fig. 1D,E), was associated with early effector differentiation of CD8+ T cells (Supplementary Table 2 of Kakaradov et al. [24]), and occurred following in vivo antigen stimulation of CD8+ T cells in murine bacterial, viral, and tumor models (Supplementary Fig. 1F, G, H), suggesting that ARS2 upregulation is a general feature of CD8+ T-cell activation. In the setting of human disease, ARS2 (*SRRT*) was significantly upregulated in CD8+ T cells from human tumors [25–27] and tumor-draining lymph nodes [28] (Fig. 1C, Supplementary Fig. 1I).

The upregulation of CBCA components, particularly ARS2, in tumor-infiltrating CD8+ T cells suggested that this critical RNA processing complex may play a role in T-cell responses. To examine the role of CBCA in human CD8+ T cells, we knocked down ARS2 via siRNA and measured the expression of six cytokines known to be involved in antitumor T-cell responses using intracellular flow cytometry. Among these cytokines, interferon gamma (IFNγ), interleukin-2 (IL-2), and tumor necrosis factor beta (TNFβ) exhibited reduced mean fluorescence intensities (MFIs) in ARS2-knockdown CD8+ T cells, while the MFIs of granzyme B and tumor necrosis factor alpha (TNFα) were increased (Supplementary Fig. 1J). Perforin was undetectable in CD8+ T cells under these experimental conditions. Further single-cell analysis of cytokine expression revealed that ARS2-knockdown T cells exhibited less polyfunctionality than did the controls (Fig. 1D). As polyfunctionality is increasingly recognized as a predictor of T-cell antitumor function [29], these data suggest that ARS2 may be important for antitumor immunity mediated by CD8+ T cells.

To further test the contribution of ARS2 to CD8+ T cells, we employed a tamoxifen-inducible ARS2 knockout (ARS2^iKO^) system because constitutive knockout of ARS2 in thymocytes results in apoptosis and failure of mature T cells to migrate to the periphery [16]. CBP80 and CBP20 knockout mice have not been described. Tamoxifen-induced ARS2 deletion had no effect on the number or viability of mature splenic T cells (Supplementary Fig. 2A, B), upregulation of T-cell activation markers (Supplementary Fig. 2C), or activation-induced cell growth (Supplementary Fig. 2D). Consistent with these findings, the frequencies of activated CD8+ T cells in the naïve (T_N_ = CD62L^hi^, CD44^−^) and effector (T_E_ = CD62L^lo^, CD44^+^) populations was unchanged by ARS2 deletion (Supplementary Figs. 1F, 2E). However, compared with control CD8+ T cells, activated ARS2^KO^ CD8+ T cells produced less IFNγ, TNFα, and IL-2 when the frequency of cells expressing each cytokine and the intensity of expression in cytokine-expressing cells was considered (Supplementary Fig. 2G, H).

To generate an antigen-specific system to test the contribution of ARS2 to CD8+ T-cell-mediated antitumor immunity in vivo, ARS2^iKO^ mice were crossed with PMEL TCR transgenic mice to generate ARS2^iKO^ CD8+ T cells that recognize the gp100_25–33_ antigen presented by H2-D^b^ on B16 melanoma cells [30]. Adoptive transfer of CD8+ T cells derived from PMEL TCR transgenic mice into B16 melanoma-bearing C57BL/6 J mice (Fig. 1E) revealed that ARS2^KO^ CD8+ T cells were unable to control tumor growth as well as ARS2^f/f^ control cells (Fig. 1F). To replicate these findings in a second adoptive therapy model, ARS2^f/f^ and ARS2^KO^ CD8+ T cells were isolated and transduced with the alpha and beta chains expressed in OT-I TCR-positive mice [31], thereby generating MHC class I-restricted OVA-specific CD8+ T cells. Equal numbers of OT-I TCR-transduced CD8+ T cells, as determined by GFP coexpression (Supplementary Fig. 2J), were adoptively transferred into mice bearing tumors derived from E.G7-OVA lymphoma cells, and parental EL4 cells were implanted in the opposite flank (Fig. 1G). Adoptively transferred OT-I TCR-transduced CD8+ ARS2^KO^ T cells did not control the growth of E.G7-OVA tumors as well as OT-I TCR-transduced CD8+ ARS2^fl/fl^ control cells did, while EL4 tumors lacking OVA expression grew at a similar rate in all mice (Fig. 1H). Importantly, IFNγ expression was upregulated in E.G7-OVA tumor-infiltrating Thy1.1^+^ CD8+ T_E_ cells in an ARS2-dependent manner (Fig. 1I). Together, these data indicate that activation-induced ARS2 upregulation supports CD8+ T-cell antitumor cytokine production and effector functions.

### CD28 signaling drives ARS2 upregulation and antitumor immunity

**Fig. 2:**
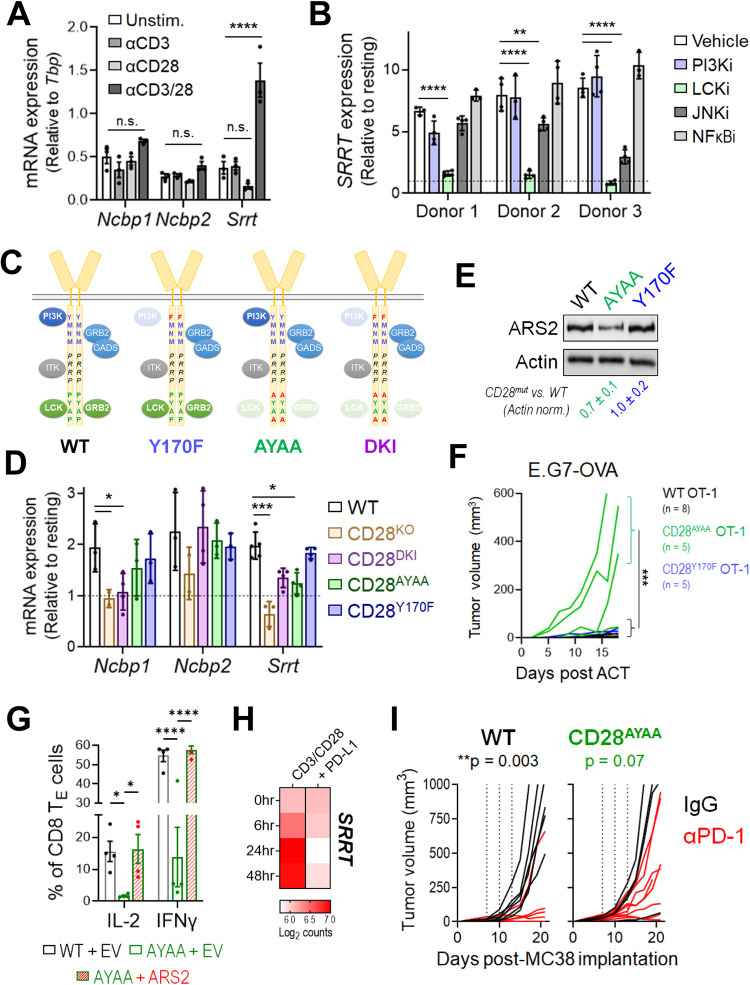
CD28 PYAP domain signaling regulates ARS2 expression and antitumor immunity **A** Expression of the mRNAs encoding the CBCA components CBP80 (*Ncbp1*), CBP20 (*Ncbp2*), and ARS2 (*Srrt*) in C57BL/6 J T cells stimulated with αCD3, αCD28, or αCD3 + αCD28 for 24 h. **B** Expression of the mRNA coding for ARS2 (*SRRT*) in CD8+ T cells isolated from healthy human donors (*n* = 3) and stimulated with αCD3 + αCD28 in the presence of the PI3K (1 μM LY294002), LCK (0.5 μM RK-24466), JNK (10 μM SP600125), or NFκB (4 μM BAY 11-7082) inhibitor. **C** Schematic depicting the CD28 intracellular signaling domain and associated signaling proteins in WT mice and CD28 mutant knock-in mice. **D** Expression of mRNAs encoding CBCA components in isolated WT C57BL/6 J, CD28 knockout, and CD28 mutant knock-in T cells stimulated with αCD3/αCD28 + rIL-2 for 24 h. **E** Representative Western blot showing ARS2 expression in isolated WT C57BL/6 J and CD28 mutant knock-in T cells stimulated with αCD3/αCD28 + rIL-2 for 72 h. The means ± SDs of the ARS2 band densities were quantified and normalized to the Actin band densities from three independent replicates (shown below the blots). **F** Growth of subcutaneous E.G7-OVA tumors following ACT with WT (black line), CD28^Y170F^ knock-in (blue line) or CD28^AYAA^ knock-in (green line) OT-1 TCR-transduced CD8+ T cells. The growth of isogenic EL4 control tumors from cells implanted into opposite flanks is shown in Supplementary Fig. 3D. **G** Rescue of IFNγ and IL-2 expression in activated CD28^AYAA^ T_E_ cells by ectopic expression of ARS2. **H** Expression of the mRNA coding for ARS2 (*SRRT*) in CD8+ T cells isolated from healthy human donors and stimulated with αCD3 + αCD28 ± recombinant PD-L1; the data are from GSE122149. **I** Growth of subcutaneous MC38 tumors in WT and CD28^AYAA^ knock-in mice treated with either 200 μg of control rat IgG2a (black lines) or αPD-1 (clone 29 F.1A12, red lines) on Days 7, 10, and 13 following tumor implantation. The bars in (**A**, **B**, **D**, **G**) indicate the means ± SDs; the dots represent biological replicates. The lines in (**F**, **I**) represent individual tumors. Differences between groups were determined by ANOVA (**A**, **B**, **D**, **G**) or mixed effects analysis (**F**, **I**). ns = not significant, *p < 0.05, **p < 0.01, ***p < 0.001, ****p < 0.0001

To begin examining the signaling requirements for CBCA upregulation, isolated T cells were stimulated with αCD3, αCD28, or both. Ligation of either CD3 or CD28 individually did not induce CBCA upregulation, while costimulation with αCD3 + αCD28 induced ARS2 mRNA (*Srrt*) expression but not *Ncbp1* or *Ncbp2* mRNA expression (Fig. 2A), indicating that ARS2 upregulation relies on TCR + CD28 costimulation, while the upregulation of other CBC components also requires signal 3 (IL-2). To further elucidate the signaling requirements for ARS2 upregulation, isolated human CD8+ T cells were stimulated with αCD3 and αCD28 in the presence of signaling inhibitors, including inhibitors of phosphoinositide 3-kinase (PI3K; 1 μM LY294002), lymphocyte-specific protein tyrosine kinase (LCK; 500 nM 7-cyclopentyl-5-(4-phenoxypenhyl)-7H-pyrrolo[2,3-d]pyrimidine-4-amine), c-Jun N-terminal Kinase (JNK; 10 μM SP600125), and Nuclear Factor Kappa B (NFκB; 4 μM BAY 11-7082). Inhibition of PI3K or NFκB had no effect on ARS2 mRNA upregulation in activated human CD8+ T cells, while JNK inhibition had a partial inhibitory effect on cells from 2 of the 3 donors. In contrast, LCK inhibition abolished ARS2 mRNA upregulation in CD8+ T cells from all three donors (Fig. 2B), indicating that TCR plus CD28 costimulation of LCK signaling was primarily responsible for the activation-induced upregulation of ARS2.

CD28 activates signaling cascades through at least three intracellular domains (ICDs) in its cytoplasmic tail [32]: the membrane proximal YMNM ICD, which activates PI3K signaling; the membrane-distal PYAP ICD, which activates signaling through LCK and growth factor receptor-bound protein 2 (GRB2); and the PRRP ICD, which is located between the YMNM and PYAP ICDs and activates signaling through interleukin-2-inducible kinase (ITK). To examine the contributions of each domain to T-cell activation-induced ARS2 upregulation, we stimulated WT, CD28 knockout [33] (CD28^KO^), CD28^AYAA^ knock-in [34], CD28^Y170F^ knock-in [35], and CD28 AYAA and Y170F double knock-in [36] (CD28^DKI^) T cells (Fig. 2C) with αCD3/αCD28 + rIL-2 to compensate for decreased IL-2 production by CD28 mutant T cells [34]. CD28 mutant T cells exhibited similar basal levels of ARS2 mRNA expression and were capable of upregulating ARS2 expression following CD28-independent stimulation with PMA and ionomycin (Supplementary Fig. 3A,B). Ex vivo stimulation of CD28-mutant T cells revealed that ARS2 upregulation required CD28 signaling through its PYAP ICD, while signaling through the CD28 PRRP ICD was required to maintain basal ARS2 mRNA expression (Fig. 2D). Limited ARS2 upregulation in CD28^AYAA^ T cells was confirmed at the protein level (Fig. 2E).

The CD28 PYAP ICD, which is necessary for ARS2 upregulation, was previously shown to be important for in vivo T-cell functions in models of allergic airway inflammation and experimental autoimmune encephalomyelitis [34–36], both of which are driven primarily by CD4+ T cells. To test whether the CD28 PYAP domain is also important for CD8+ T-cell-mediated antitumor immunity, OT-I TCR-transduced WT, CD28^AYAA^ knock-in, and CD28^Y170F^ knock-in CD8+ T cells (Supplementary Fig. 3C) were adoptively transferred into E.G7-OVA/EL4 tumor-bearing mice, as shown in Fig. 1I. Similar to adoptive transfer of ARS2^KO^ OT-I TCR-transduced CD8+ T cells, adoptive transfer of OT-I TCR-transduced CD28^AYAA^ CD8+ T cells failed to control E.G7-OVA tumor growth as well as WT control cells did, while OT-I TCR-transduced CD28^Y170F^ CD8+ T cells demonstrated an antitumor function similar to that of WT control cells (Fig. 2F and Supplementary Fig. 3D). IFNγ and IL-2 expression in CD28^AYAA^ CD8 effector T cells was also reduced compared to that in WT and CD28^Y710F^ knock-in T cells (Supplementary Fig. 3E) and was rescued by exogenous overexpression of ARS2 (Fig. 2G), establishing the existence of a CD28-ARS2 axis capable of inducing effector cytokine expression and CD8+ T-cell-mediated antitumor immunity.

The suppressive function of the PD-1 checkpoint in CD8+ T cells has emerged as a salient therapeutic target in several types of cancer [37]. T-cell surface-expressed PD-1 functions by recruiting phosphatases to immunological synapses, resulting in the dephosphorylation of critical T-cell signaling molecules, including CD28 [38]. As such, CD28 has been proposed to be a primary target of PD-1 in its suppression of T-cell activation [39, 40], leading us to test whether PD-1 ligation can inhibit CD28-dependent upregulation of ARS2 in activated T cells. Analysis of public data (GSE122149 [41]) confirmed that activation of isolated human CD8+ T cells resulted in upregulation of ARS2 expression and further demonstrated that PD-1 ligation abolished ARS2 mRNA upregulation (Fig. 2H). This finding raised the intriguing possibility that PD-1 may inhibit not only CD28-mediated PI3K signaling [39] but also signaling through the CD28 PYAP ICD. We therefore reasoned that loss of CD28 PYAP ICD-mediated signaling would render T cells nonresponsive to αPD-1 checkpoint inhibitor therapy. Using immune-responsive MC38 colon carcinoma cells implanted in WT C57BL/6 mice and CD28^AYAA^ mice, we found that disruption of CD28 PYAP ICD-mediated signaling severely limited the antitumor efficacy of anti-PD-1 antibody therapy (Fig. 2I), indicating that CD28 PYAP ICD-mediated downstream signaling and effector proteins such as ARS2 may be relevant to cancer immunotherapy.

### The CD28-ARS2 axis regulates T-cell activation-induced alternative splicing

**Fig. 3:**
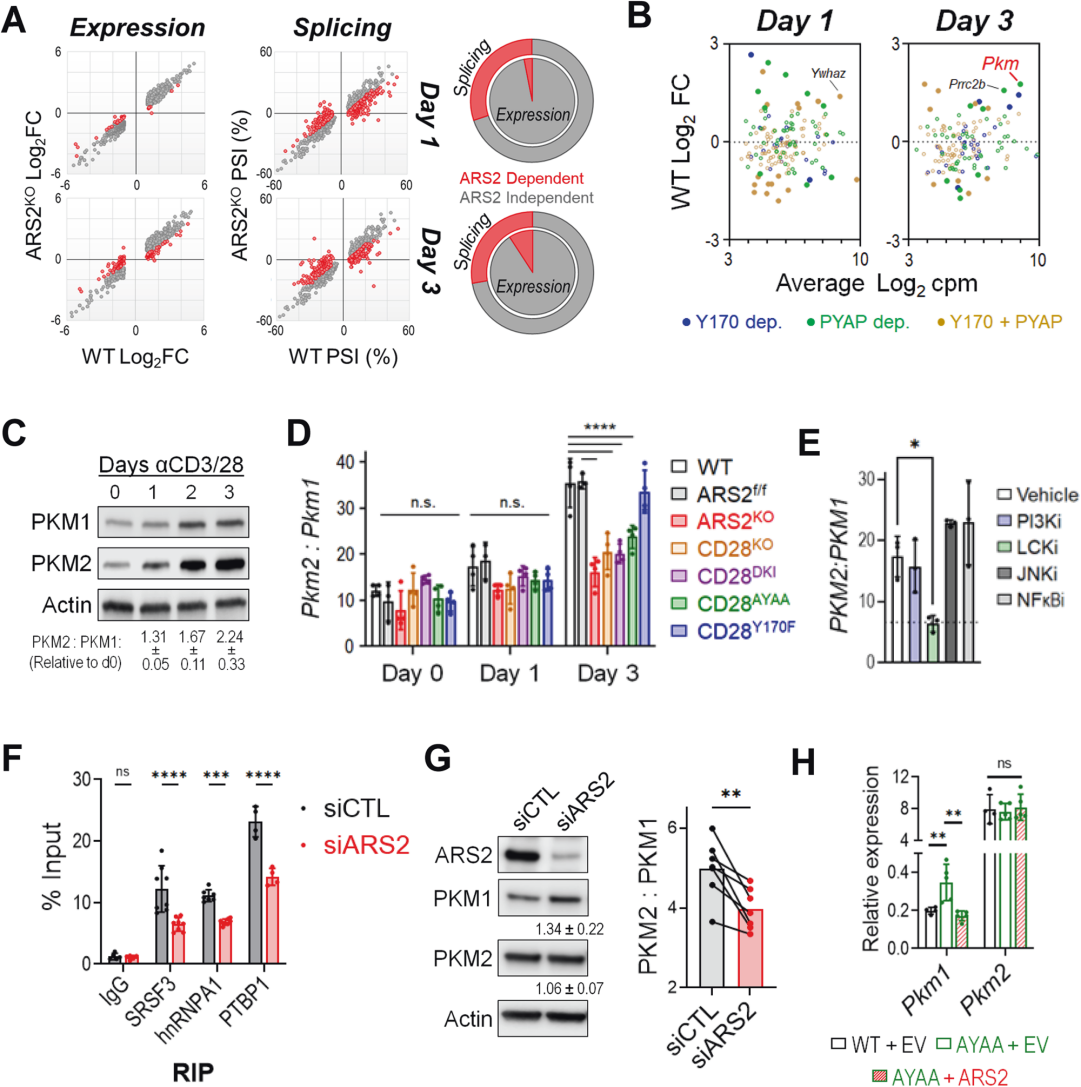
T-cell activation-induced alternative splicing of *PKM* is regulated by the CD28-ARS2 axis. **A** Effect of ARS2 deletion on T-cell activation-induced gene expression (left) or alternative splicing (right) on Day 1 (top) and Day 3 (bottom) following stimulation with αCD3/αCD28 + rIL-2. The red dots indicate significant differences between activated WT and ARS2^KO^ T cells. The pie charts depict the contribution of ARS2 to differential gene expression (inner pie) and alternative splicing (outer pie) induced by T-cell activation. **B** Dot plot showing fold changes in the expression of genes whose alternative splicing was found to be ARS2 and CD28 dependent on Day 1 (left) or Day 3 (right) of T-cell activation in activated T cells compared with resting T cells. The dots are color coded based on CD28 signaling domain dependence. The filled dots represent DEGs in activated cells relative to resting cells. **C** Western blot analysis of PKM1 and PKM2 protein expression in human T cells stimulated with αCD3/αCD28-coated microbeads; representative of four normal donors. **D** *Pkm2*-to-*Pkm1* ratio, as determined by qRT‒PCR, in WT, ARS2^f/f^, ARS2^KO^, CD28^KO^, CD28^DKI^ knock-in, CD28^AYAA^ knock-in, and CD28^Y170F^ knock-in T cells following activation with αCD3/αCD28 + rIL-2 for the indicated number of days. Total *Pkm* expression is shown in Supplementary Fig. 4E. **E** *PKM2*-to-*PKM1* ratio, as determined by qRT‒PCR, in human T cells activated for 3 days with αCD3/αCD28-coated microbeads in the presence of the indicated inhibitors, as described in Fig. 2B. **F** Binding of splicing factors to *PKM* pre-mRNA, as determined by RNA immunoprecipitation (RIP) followed by qRT‒PCR, in control siRNA- or ARS2 siRNA-transfected human T cells activated with αCD3/αCD28-coated microbeads for 3 days. **G** Western blot showing the expression of PKM1 and PKM2 in control compared with ARS2-knockdown human T cells on Day 3 of activation. The values below the blots are the mean expression levels in cells with ARS2 knockdown relative to those in control cells ± the SD values from 7 healthy donors. The bar graph on the right shows the average PKM2-to-PKM1 protein ratio, with the connected dots representing individual donors. Differences between groups were determined by paired *t* tests. **H** Expression of *Pkm1* and *Pkm2* on Day 3 of activation in empty vector (EV)-transfected WT T cells compared to CD28^AYAA^ knock-in T cells transfected with either EV or ARS2. The bars in (**D**, **E**, **F**, **H**) indicate the means ± SDs; the dots represent biological replicates. Differences between groups were determined by ANOVA. ns = not significant, *p < 0.05, **p < 0.01, ***p < 0.001, ****p < 0.0001

To determine how ARS2 affects T-cell function, we performed RNA sequencing on WT and ARS2^KO^ T cells immediately following isolation (Day 0) and following stimulation with αCD3/αCD28 microbeads + rIL-2 for 24 h (Day 1) or 72 h (Day 3). Surprisingly, the vast majority of differentially expressed genes (DEGs) in activated T cells were unaffected by ARS2 deletion (Fig. 3A and Supplementary Tables S1–S3). However, PSI-Sigma [42] analysis revealed that between one-quarter and one-third of alternative splicing events induced by T-cell activation were disrupted in ARS2^KO^ T cells (Fig. 3A and Supplementary Tables S4, S5). Less common splicing events, including “alternative 5′ splice site utilization”, “alternative 3′ splice site utilization” and “mutually exclusive splicing”, were particularly sensitive to ARS2 deletion (Supplementary Fig. 4A). Importantly, these data are the first to demonstrate a regulatory role for ARS2 in alternative splicing despite the identification of multiple splicing factors in previous ARS2 interactome studies [13, 14, 43].

Since functional studies revealed that the CD28-ARS2 pathway is activated by CD28 PYAP ICD-mediated signaling, we repeated the RNA-seq analysis of T-cell activation-induced alternative splicing using CD28-mutant T cells. We observed marked overlap between T-cell activation-induced alternative splicing events regulated by CD28 ICDs and ARS2, with more than half of the ARS2-dependent splicing events also requiring intact CD28 PYAP ICD-mediated signaling (Supplementary Fig. 4B and Supplementary Tables S8, S9). In contrast, the vast majority of ARS2-independent splicing events occurred normally in the absence of CD28 signaling (Supplementary Fig. 4B, C).

### Alternative splicing of *PKM* is regulated by the CD28-ARS2 axis during T-cell activation

We reasoned that splicing events in transcripts that were both highly expressed and upregulated by T-cell activation would be the most likely to have biological consequences. We therefore examined transcripts that underwent ARS2- and CD28-dependent alternative splicing in resting compared with activated WT T cells (Fig. 3B and Supplementary Tables S6, S7). Several interesting transcripts were identified via this analysis and included *Pkm*, the transcript encoding pyruvate kinase (PKM), the final enzyme of glycolysis. PKM is expressed as two isoforms that result from mutually exclusive alternative splicing events; PKM1 is expressed mainly in quiescent cells, while PKM2 has been linked to the Warburg effect in tumor cells [44]. Activation of human T cells led to a large increase in PKM2 protein expression but had a relatively limited effect on PKM1 (Fig. 3C), consistent with changes in *PKM* alternative splicing to favor the inclusion of exon 10. Similarly, the PKM2 protein was enriched 3 days after antigenic stimulation of OT-I T cells in vitro or in vivo (Tables S1, S2 in ref. [45]). Through an established RT‒PCR/restriction digestion method [46, 47], we confirmed enrichment of *PKM2* mRNA relative to *PKM1* mRNA in activated human T cells (Supplementary Fig. 4D). Isoform-specific qRT‒PCR showed that *Pkm2* expression was preferentially induced in activated mouse T cells on Day 3 in an ARS2- and CD28 PYAP ICD-dependent manner (Fig. 3D), confirming RNA-seq results. Importantly, the total *Pkm* mRNA level in activated ARS2^KO^ and CD28^AYAA^ T cells was equivalent to that in activated WT T cells (Supplementary Fig. 4E), further indicating that the CD28-ARS2 axis primarily affects *Pkm* alternative splicing. The results of signaling inhibitor experiments supported this finding, as the same LCK inhibitor that blocked ARS2 upregulation (Fig. 2B) also reduced the ratio of *PKM2* to *PKM1* mRNA in activated human T cells (Fig. 3E).

### ARS2 facilitates the association of splicing factors with *PKM* pre-mRNA and the suppression of *PKM1* expression

The alternative splicing of *PKM* exons 9 and 10 is regulated by the RNA binding proteins hnRNPA1/A2, PTBP1, and SRSF3 [9]. Published RNA interactome data indicate that ARS2, SRSF3, hnRNPA1, and PTBP1 all bind to the *PKM* transcript in human cell lines [48–50] (Supplementary Fig. 4F). The results of RNA immunoprecipitation (RIP) followed by qRT‒PCR using intron–exon junction spanning primer sets to detect *Pkm* pre-mRNA confirmed these interactions in the murine hematopoietic cell line FL5.12 (Supplementary Fig. 4G). ARS2 binding to *Pkm* pre-mRNA was further confirmed in mouse and human T cells (Supplementary Fig. 4H,I) and was disrupted by CBP80 knockdown (Supplementary Fig. 4J), indicating that the association of ARS2 with *Pkm* pre-mRNA is mediated by the CBC.

Since the primary function of CBC-bound ARS2 is to recruit RNA processing factors to nascent transcripts [12, 13, 18, 43, 48], we asked whether ARS2 affects splicing factor association with *Pkm* pre-mRNA and found that depletion of ARS2 in primary T cells or cell line models resulted in substantial reductions in the binding of SRSF3, hnRNPA1 and PTBP1 to *Pkm* pre-mRNA (Fig. 3F and Supplementary Fig. 4K). These data suggest a model in which ARS2 upregulation mediated by CD28 supports the recruitment of splicing factors to *Pkm* pre-mRNA, thereby favoring PKM2 expression in activated T cells. However, the net result of the reduced splicing factor binding to *PKM* pre-mRNA has not been determined. The increase in PKM1 protein expression in the absence of a change in PKM2 protein expression in activated ARS2 knockdown human T cells (Fig. 3G) and the increased *Pkm1* mRNA expression in the absence of a change in *Pkm2* mRNA expression in activated CD28^AYAA^ mouse T cells (Fig. 3H) indicated that the primary effect of the CD28-ARS2 axis on *Pkm* splicing is inhibition of exon 9 inclusion. Ectopic expression of ARS2 in activated CD28^AYAA^ T cells reduced *Pkm1* expression to WT levels without affecting *Pkm2* expression (Fig. 3H), further demonstrating that the CD28-ARS2 axis suppresses *Pkm* exon 9 inclusion during T-cell activation.

### PKM2 supports CD8+ T-cell-mediated antitumor immunity and metabolic reprogramming

**Fig. 4:**
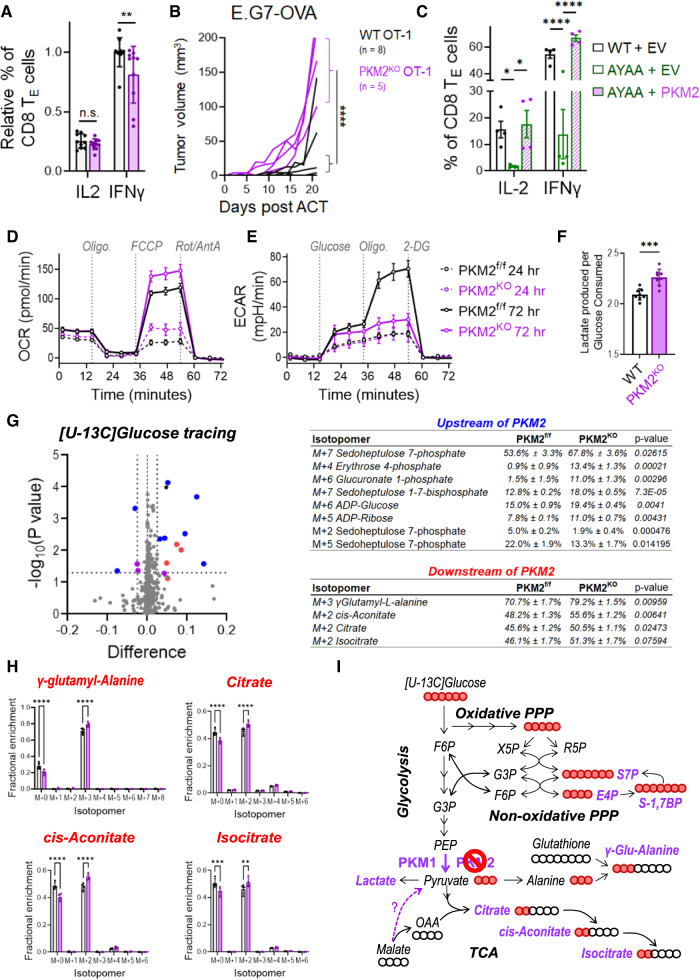
Influence of PKM2 on CD8+ T-cell antitumor function and metabolic reprogramming. **A** IFNγ and IL-2 expression in activated PKM2^f/f^ and PKM2^KO^ T_E_ cells (CD8^+^CD44^+^CD62L^−^) after 72 h of ex vivo activation. **B** Growth of subcutaneous E.G7-OVA tumors following ACT with WT (black line) or PKM2^KO^ (purple line) OT-1 TCR-transduced CD8+ T cells. The growth of isogenic EL4 control tumors from cells implanted into opposite flanks is shown in Supplementary Fig. 5G. **C** Rescue of IFNγ and IL-2 expression in activated CD28^AYAA^ T_E_ cells by ectopic expression of PKM2. **D** Seahorse Mito Stress Tests were performed on PKM2^f/f^ (black lines) and PKM2^KO^ (purple lines) T cells 24 h (dashed lines) and 72 h (solid lines) after activation with αCD3/αCD28-coated microbeads + rIL-2. **E** Seahorse Glycolysis Stress Tests were performed as described in (**D**). **F** The ratio of lactate produced to glucose consumed was measured in the culture medium of WT and PKM2^KO^ T cells on day three of activation. **G** Volcano plot depicting differences in the fractional enrichment of metabolite isotopomers labeled with [U-^13^C] glucose in Day 3 activated PKM2^KO^ compared with PKM2^f/f^ CD8+ T cells. The blue dots represent differentially labeled metabolites upstream of PKM2, the red dots represent metabolites downstream of PKM2, and the purple dots represent differentially labeled nucleotides. The tables on the right show the fractional enrichment of differentially labeled metabolite isotopomers upstream and downstream of PKM2. **H** Increased ^13^C-glucose labeling of isotopomers of metabolites downstream of PKM2 in Day 3 activated PKM2^KO^ CD8+ T cells. **I** Diagram of metabolic pathways altered in Day 3 activated PKM2^KO^ CD8+ T cells. The abundances of the indicated isotopomers of the metabolites shown in purple were significantly increased in Day 3 activated PKM2^KO^ CD8+ T cells. The solid red balls represent ^13^C carbons, and the empty balls represent ^12^C carbons. The bars in (**A**, **C**, **F**, **H**) indicate the means ± SDs; the dots represent biological replicates. The lines in (**B**) represent individual tumors. The Seahorse plots in (**D**, **E**) show representative results from experiments repeated at least 3 times. Differences between groups were determined by ANOVA (**A**, **C**, **F**, **H**) or mixed effects analysis (**B**). ns = not significant, *p < 0.05, **p < 0.01, ***p < 0.001, ****p < 0.0001

To directly test the effects of *Pkm* alternative splicing on activated CD8+ T cells, we generated *Pkm2* inducible knockout mice (PKM2^iKO^) by crossing *Pkm2*^f/f^ mice [51] with CreERT2-expressing mice [52]. Tamoxifen administration to PKM2^iKO^ mice led to loss of PKM2 in peripheral T cells and compensatory upregulation of PKM1 (Supplementary Fig. 5A, B). PKM2 knockout did not alter the number or frequency of splenic T cells (Supplementary Fig. 5C,D) or reduce their ability to express activation markers following stimulation with αCD3/αCD28 + rIL-2 (Supplementary Fig. 4E). However, PKM2^KO^ CD8 T_E_ cells were unable to fully induce IFNγ expression or control tumor growth (Fig. 4A, B and Supplementary Fig. 5F,G), similar to ARS2^KO^ and CD28 mutant T cells (Fig. 1, 2). Furthermore, ectopic expression of PKM2 rescued effector cytokine production in CD28^AYAA^ T cells (Fig. 4C), supporting the concept that ARS2-directed alternative splicing of *Pkm* could be a mechanism by which the CD28-ARS2 axis influences T-cell-mediated antitumor immunity. Unfortunately, the technical limitations of retroviral PKM2 overexpression in T cells lacking ARS2 limited our ability to test whether PKM2 could also rescue the defects observed in ARS2^KO^ T cells (Supplementary Fig. 5H).

Since PKM is the final rate-limiting enzyme in the glycolysis pathway and since glycolytic flux has been shown to regulate T-cell effector cytokine expression [5, 53, 54], we next examined the effects of PKM2 knockout on T-cell metabolism. Consistent with the increased enzymatic activity of PKM1 relative to PKM2 [9], activated PKM2^KO^ T cells exhibited an increased respiratory capacity compared to that of T cells from littermate control mice (Fig. 4D). No difference in the extracellular acidification rate (ECAR), a surrogate marker for aerobic glycolysis, was observed in PKM2^KO^ T cells 24 h after activation (Fig. 4E, dashed lines), a time point at which a relatively minor change in the ratio of PKM2 to PKM1 was observed (Fig. 3C, D). However, PKM2^KO^ T cells failed to fully activate their glycolytic reserve – the ability to use glycolysis when mitochondrial metabolism is inhibited – by Day 3 of activation (Fig. 4E, solid lines), a time point at which the preference for PKM2 expression in WT T cells was pronounced (Fig. 3C, D). Decreased ECAR did not result from a reduction in Warburg metabolism in PKM2^KO^ CD8+ T cells, as they secreted more lactate per molecule of glucose consumed than did WT CD8+ T cells (Fig. 4F). Overall, these data indicate that PKM2 endows T cells with metabolic flexibility to increase aerobic glycolysis when mitochondrial metabolism is limited, as is often the case in hypoxic tumor microenvironments [55].

### PKM2 limits glucose flux into the TCA cycle and nonoxidative arm of the PPP

To further characterize the influence of PKM2 on CD8+ T-cell metabolism, we performed [U-^13^C] glucose tracing three days after ex vivo activation of CD8+ T cells and compared glucose-labeled isotopomers between activated PKM2^KO^ T cells and PKM2^f/f^ T cells (Fig. 4G). Prior work showed that the expression of PKM2 in tumor cells results in the accumulation of glycolytic intermediates that can be used for biosynthesis [9]. In contrast, we found that glucose carbon enrichment in several intermediate metabolites upstream of PKM2, including several fully labeled intermediates of the nonoxidative pentose phosphate pathway (PPP), was enhanced in PKM2^KO^ T cells (Fig. 4G and Supplementary Fig. 5I–K). Among these metabolites, sedoheptulose 7-phosphate (S7P) exhibited a labeling pattern (increased M+7, decreased M+2 and M+5), consistent with decreased transketolase activity (Supplementary Fig. 5I).

Increased glucose carbon enrichment in PKM2 downstream metabolites indicated that the switch in PKM isoform expression from a predominance of PKM2 to PKM1 increased glucose flux into pyruvate and subsequently into the tricarboxylic acid (TCA) cycle (Fig. 4H, I), although technical challenges precluded accurate measurement of pyruvate in these experiments. Additional data suggested enhanced glucose flux into pyruvate and subsequently alanine, as PKM2^KO^ T cells exhibited an increased abundance of the M+3 isotopomer of the dipeptide γ-glutamyl-alanine (derived from alanine and glutathione) in the absence of glucose carbon labeling of glutathione (Fig. 4H, I and Supplementary Fig. 5L). Taken together, our metabolic characterization of PKM2^KO^ CD8+ T cells indicated that PKM2 reduces glucose carbon flux into pyruvate and downstream metabolites, resulting in enhanced metabolic flexibility in activated CD8+ T cells.

### The CD28-ARS2-PKM2 axis endows activated CD8+ T cells with metabolic flexibility

**Fig. 5:**
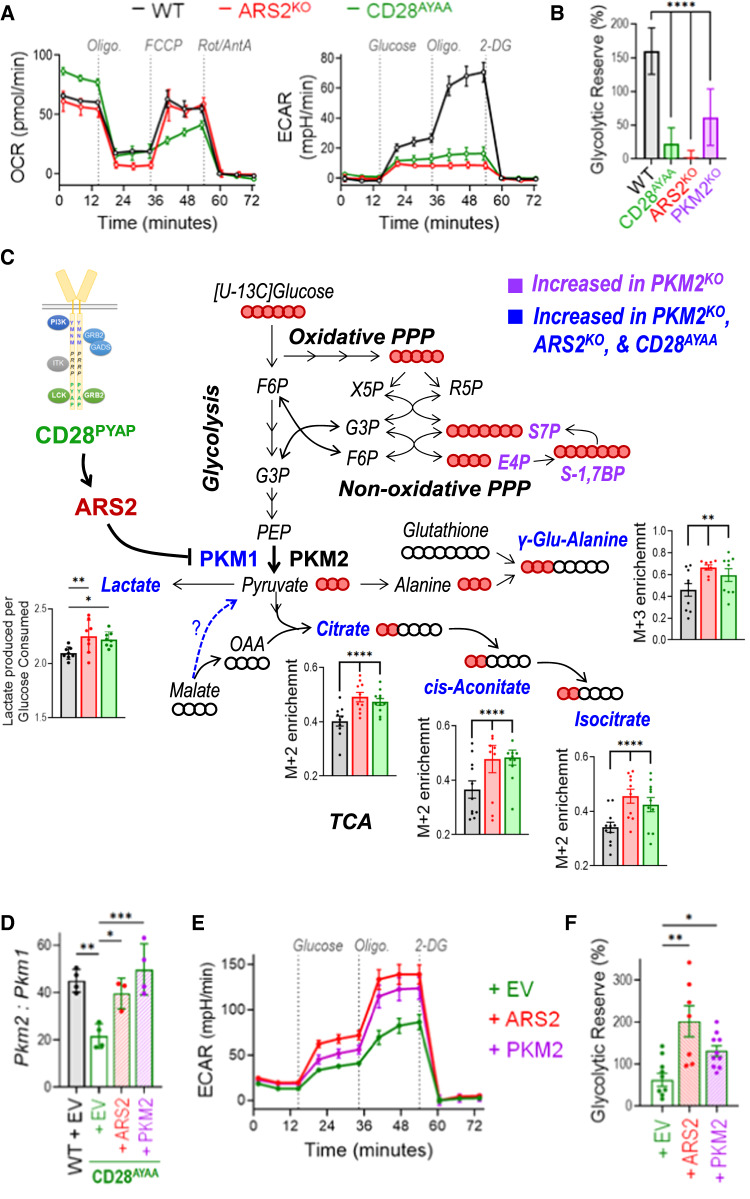
The CD28-ARS2-PKM2 axis imparts CD8+ T cells with metabolic flexibility. **A** Seahorse Mito (left) and Glycolysis (right) Stress Tests were performed on WT (black lines), ARS2^KO^ (red lines), and CD28^AYAA^ knock-in (green lines) T cells 72 h after activation with αCD3/αCD28-coated microbeads + rIL-2. **B** Quantification of the glycolytic reserve (maximum ECAR ÷ glucose-induced ECAR) in Day 3 activated T cells of the indicated genotypes. **C** Diagram of metabolic pathways influenced by the CD28-ARS2-PKM2 axis in Day 3 activated CD8+ T cells; the bar graphs show significant changes in ARS2^KO^ (red bars) and CD28^AYAA^ knock-in (green bars) cells. The abundances of the indicated isotopomers of the metabolites shown in blue were significantly increased in Day 3 activated CD28^AYAA^ knock-in, ARS2^KO^, and PKM2^KO^ CD8+ T cells, while those in purple were only increased in PKM2^KO^ CD8+ T cells. The solid red balls represent ^13^C carbons, and the empty balls represent ^12^C carbons. **D** *Pkm2*-to-*Pkm1* ratio on Day 3 after activation in WT T cells transfected with empty vector (EV) compared to CD28^AYAA^ knock-in T cells transfected with EV, ARS2, or PKM2. **E** Seahorse Glycolysis Stress Tests were performed on Day 3 activated CD28^AYAA^ knock-in T cells transfected with EV, ARS2, or PKM2. **F** Quantification of the glycolytic reserve in Day 3 activated CD28^AYAA^ knock-in T cells transfected with EV, ARS2, or PKM2. The bars in (**B**, **C**, **D**, **F**) indicate the means ± SDs; the dots represent biological replicates. The Seahorse plots in (**A**, **E**) show representative results of experiments repeated at least 3 times. Differences between groups were determined by ANOVA. ns = not significant, *p < 0.05, **p < 0.01, ***p < 0.001, ****p < 0.0001

Because the physiological regulation of the ratio of PKM2 to PKM1 by the CD28-ARS2 axis was minimal compared to the dramatic change resulting from PKM2 knockout (Supplementary Fig. 6A), we sought to determine whether the CD28-ARS2-dependent changes in *Pkm* alternative splicing affect metabolic reprogramming in activated CD8+ T cells. Seahorse assays demonstrated inconsistent perturbations in oxidative metabolism among Day 3 activated ARS2^KO^, CD28^AYAA^, and PKM2^KO^ T cells (Figs. 4D, 5A). In contrast, Seahorse ECAR measurements revealed a severely compromised glycolytic reserve in Day 3 activated ARS2^KO^, CD28^AYAA^, and PKM2^KO^ T cells (Figs. 4E, 5A,B). Further metabolic characterization revealed largely overlapping changes in metabolites downstream of PKM in Day 3 activated ARS2^KO^, CD28^AYAA^, and PKM2^KO^ CD8+ T cells, including increased lactate secretion per molecule of glucose consumed, an increased abundance of the M+3 isotopomer of γ-glutamyl-alanine in the absence of glucose carbon labeling of glutathione, and increased M+2 labeling of the TCA cycle intermediates citrate, cis-aconitate, and isocitrate (Fig. 5C and Supplementary Fig. 6B). In contrast, glucose labeling of PPP metabolites was not affected by CD28 mutation or loss of ARS2 (Fig. 5C and Supplementary Fig. 6C–E), indicating that the physiological perturbation of the ratio of PKM2 to PKM1 may not be sufficient to alter glucose flux through the nonoxidative PPP. Together, the results of these metabolomic analyses revealed that physiological regulation of PKM isoform expression via the CD28-ARS2 axis impacts glucose carbon flux in Day 3 activated T cells and allows the activation of aerobic glycolysis in these cells when mitochondrial oxidative metabolism is inhibited.

To definitively test the contribution of ARS2 and PKM2 to the CD28-driven reprogramming of glucose metabolism, we revisited the overexpression experiments in which ectopic expression of ARS2 or PKM2 restored IFNγ expression in activated CD28^AYAA^ mutant CD8 effector T cells to WT levels (Figs. 2G, 4C). Despite having distinct effects on PKM isoform expression in activated CD28^AYAA^ mutant T cells - ARS2 overexpression decreased *Pkm1* expression (Fig. 3H), while PKM2 overexpression increased *Pkm2* expression (Supplementary Fig. 6G) - ectopic expression of either ARS2 or PKM2 in activated T cells led to restoration of the WT *Pkm2*-to-*Pkm1* ratio (Fig. 5D). Restoration of the WT *Pkm2*-to-*Pkm1* ratio was associated with an increased ECAR (Fig. 5E) and increased glycolytic reserve (Fig. 5F) in activated CD28^AYAA^ T cells. These data confirm that ARS2 and PKM2 are downstream effectors of CD28 that endow activated T cells with metabolic flexibility.

## Discussion

To provide protective antitumor immunity, CD8+ T cells must undergo dramatic transcriptional and metabolic reprogramming to permit their effector differentiation and cytotoxic function. While the role of transcription factors has been extensively characterized, much less is known about how cotranscriptional processes contribute to CD8+ T-cell metabolic reprogramming and acquisition of effector properties. In this study, we aimed to determine how a key cotranscriptional regulator, the ARS2-bound nuclear 5′-methylguanosine cap-binding complex (CBCA), induced by T-cell activation contributes to the CD8+ T-cell antitumor response. In doing so, we uncovered a novel CD28-driven mechanism by which the CBCA directs the alternative splicing of *PKM*, allowing activated CD8+ T cells to acquire flexibility in their use of glucose to fuel their effector functions.

Previous studies defining the roles of alternative splicing in T cells have largely focused on CD4+ T cells, where splicing of transcripts encoding surface receptors (*CD45, CD44*), signaling molecules (*MALT1*) and transcription factors (*FOXP3*) all contribute to CD4 differentiation and/or function [56]. Additionally, splicing factors, including hnRNPLL and CELF2, were shown to be upregulated by TCR and/or CD28 coreceptor signaling in CD4+ T cells and to influence T-cell activation-induced alternative splicing [57, 58]. A recent study revealed that alternative splicing patterns were similar between CD4+ and CD8+ T cells [59], yet the regulatory and functional implications of alternative splicing in CD8+ T cells have remained largely unexplored.

ARS2 has emerged over the past decade as a critical intermediary physically linking the CBC to RNA maturation and degradation pathways long known to rely on RNA capping and CBC binding [11]. To the best of our knowledge, the data in the present study are the first to demonstrate that ARS2 regulates the CBC-dependent process of alternative pre-mRNA splicing. The RNA-seq and qRT‒PCR data, coupled with data demonstrating that ARS2 recruits hnRNPA1, PTBP1, and SRSF3 to *PKM* pre-mRNA to suppress *PKM* exon 9 inclusion, provide compelling mechanistic evidence that ARS2 facilitates alternative splicing during T-cell activation. Data further indicate that ARS2 regulates additional splicing events during T-cell activation, potentially by recruiting PTBP1, hnRNPA1, SRSF3, and/or other splicing factors to nascent transcripts. Further examination of ARS2-directed alternative splicing events, or potentially other ARS2-directed RNA maturation events, may reveal additional ways in which the CBCA regulates CD8+ T-cell activation and function. Furthermore, it is unclear whether the newly identified role of ARS2 in facilitating alternative splicing is limited to activated T cells or is broadly relevant.

The results of knockout experiments established that one product of ARS2-directed alternative splicing, PKM2, allows activated CD8+ T cells to fully induce glycolysis, display metabolic flexibility, produce high levels of IFNγ, and mediate efficient antigen-specific control of tumor growth in mice. Prior studies showed that IFNγ expression was similarly reduced in CD4+ T cells by PKM2 inhibition [60] or knockout [61]; however, in this context, PKM2 appeared to have a more dominant role in directing differentiation via direct effects on gene transcription [6–8]. The nonmetabolic role of PKM2 in directly driving transcription remains controversial [9], and experiments to tease out the metabolic functions from the nonmetabolic functions of PKM2 are quite challenging, especially in light of the growing body of evidence indicating that metabolic perturbations often directly influence the function of epigenetic enzymes [62]. Our data indicate that PKM2 drives IFNγ production in CD8 effector T cells posttranscriptionally (Supplementary Fig. 5F) and, therefore, likely by altering flux through glycolysis, resulting in relief of GAPDH-mediated repression of *Ifng* mRNA translation [5]. However, whether the nonmetabolic functions of PKM2 contribute to CD8+ T-cell antitumor activity remains to be determined.

Recent studies have demonstrated the importance of CD28 in glycolysis as well as in antitumor effector differentiation and function within the tumor microenvironment [28, 63, 64]. Our recent finding that the CD28 PYAP ICD is necessary for increased glycolysis in T cells during the first 24 h of activation [65] added to the findings of earlier studies that implicated PI3K activation by CD28 in the upregulation of GLUT1 expression and glycolysis over the same 24-h activation period [4, 66], indicating that the signaling initiated by the membrane-proximal YMNM domain and membrane-distal PYAP domain of CD28 may cooperate to induce glycolytic metabolism during T-cell priming. Intuitively, metabolic states should evolve to meet cellular demands, as primed CD8+ T cells first increase in size (blastogenesis) and then undergo proliferative expansion and differentiation into effector or memory populations. The data presented here show that CD28 PYAP ICD-mediated signaling further induces glycolysis and allows the establishment of a glycolytic reserve after initial T-cell priming. Glycolytic reserve indicates the ability of T cells to rapidly induce glycolysis when mitochondrial metabolism is inhibited, as often occurs in the hostile tumor microenvironment due to hypoxia and/or nutrient deprivation. As such, our finding that the CD28-ARS2-PKM2 axis endows the metabolic flexibility needed for optimal CD8+ T-cell-mediated antitumor immunity in mice is consistent with recent reports demonstrating that CD28-stimulated glucose metabolism enhances effector properties and the checkpoint blockade response of tumor-infiltrating T cells in cancer patients [63, 64, 67]. This raises the intriguing possibility that developing strategies to reinforce the CD28-ARS2-PKM2 axis in tumor-targeted CD8+ T cells could improve the efficacy of cancer immunotherapy.

## Methods

### Mice

Experiments involving mice were performed at the Laboratory Animal Shared Resource of Roswell Park Comprehensive Center following approved IACUC protocols. Wild-type C57BL/6 (000664) mice were purchased from The Jackson Laboratory. CD28^AYAA^ knock-in (CD28^tm1Jmg^), CD28^Y170F^ knock-in (CD28^tm2.1Jmg^), CD28^DKI^ (CD28^tm2.2Jmg^), CD28^KO^ (CD28^tm1Mak^/J), and ARS2^iKO^ mice were generated as previously described [16, 37–40]. PKM2^iKO^ mice were generated by crossbreeding PKM2^f/f^ mice (B6;129S-Pkmtm1.1Mgvh/J; The Jackson Laboratory stock #024048) with R26-CreERT2 mice (B6.129-Gt(ROSA)26Sortm1(cre/ERT2)Tyj/J; The Jackson Laboratory stock #008463). To induce ARS2 or PKM2 deletion, 6‒12-week-old mice were intraperitoneally injected with 100 µg tamoxifen (Sigma, 10540-29-1) daily for 5 days followed by 2 days of rest. Sex-matched littermates that were negative for CreERT2 expression also received tamoxifen and were used as controls. To generate ARS2^iKO^ mice expressing the transgenic tumor antigen-specific PMEL TCR, ARS2^iKO^ mice were crossed with PMEL mice (B6.Cg-Thy1a/Cy Tg(TcraTcrb)8Rest/J; The Jackson Laboratory stock #005023). All the mice were 6–12 weeks old at the start of the experiments. Both male and female mice were used, and no sex-specific differences were observed.

### Tumor models

Six- to 12-week-old male mice were subcutaneously implanted with 1 × 10^5^ EL4 cells in one flank and 1 × 10^6^ EG7-OVA cells in the other flank three days prior to adoptive transfer of 1 × 10^6^ OT-I TCR-transduced (as determined by the % of GFP+ expression) T cells isolated from mice of the indicated genetic background. Similarly, 0.5 × 10^6^ B16 melanoma cells were implanted in the flanks of female mice 4 days prior to adoptive transfer of 1 × 10^6^ freshly isolated ARS2-expressing or ARS2-knockout PMEL TCR transgenic T cells. For all the models, tumor measurements were performed every 2–3 days using calipers, and tumor volumes were calculated as ((L^2)/2)*W. Littermates of the same sex were randomly assigned to the experimental groups.

### Cell culture

All cell culture was conducted in tissue culture incubators maintained at 37 °C and 5% CO_2_. B16 melanoma, FL.12, FL5.12.xL, EL4, and EG7-OVA (from Dr. Ruea-Yea Huang) cells were maintained in RPMI 1640 medium supplemented with 10% FBS (Corning), 10 U/mL pen/strep, 2 mM L-glutamine, 50 μM β-mercaptoethanol, and 10 mM HEPES (Thermo Fisher). For EG7-OVA cells, the culture medium also contained 0.4 mg/ml G418 (Selleck Chemicals). For FL5.12 and FL5.12.xL cells, the medium also contained 0.35 ng/mL IL-3. MC38 cells (from Dr. Sandra Gollnick) were grown in DMEM supplemented with 10% FBS, pen/strep, L-glutamine, HEPES, 0.1 mM nonessential amino acids and 1 mM sodium pyruvate (Thermo Fisher). All cell lines were regularly tested using the MycoAlert Mycoplasma Detection Kit (Lonza).

### Mouse T-cell isolation/activation

Peripheral T cells were isolated from the spleens of 6–12-week-old mice via negative selection using the EasySep Mouse T-cell Isolation Kit or the EasySep Mouse CD8^+^ T-cell Isolation Kit (STEMCELL) and activated with anti-CD3/CD28 beads (Miltenyi T-cell Activation/Expansion Kit, mouse) at a 1:1 bead:cell ratio in the presence or absence of 25 U/mL recombinant murine IL-2 (BioLegend), as noted in the figure legends. Activated T cells were maintained at a density of 1 × 10^6^ cells/mL, and the size of the cells was measured using a Z2 Coulter Counter (Beckman).

### Human T-cell isolation/activation

T cells were isolated from peripheral blood from healthy donors collected in EDTA-coated tubes (Vacutainer, BD Biosciences) or from discarded Trima cones obtained from platelet donors in accordance with protocols approved by the Institutional Review Board (IRB) of Roswell Park Comprehensive Cancer Center, Buffalo, NY, following federal and state laws and in compliance with the Declaration of Helsinki. T-cell isolation was performed using MACSxpress Pan T-cell Isolation Kits (Miltenyi Biotec). Freshly isolated T cells (1 × 10^6^ cells/mL) were stimulated with anti-CD3/CD28 Dynabeads (Thermo Fisher Scientific) or T-Cell TransAct (Miltenyi Biotec) in RPMI 1640 medium supplemented with 5% HI-FBS, HEPES, NEAA, sodium pyruvate, and penicillin/streptomycin. For experiments using chemical inhibitors, each inhibitor was added separately to T cells immediately prior to activation, after which the cells were maintained in culture medium throughout the experiment. The following inhibitors used in the described studies were purchased from Cayman Chemicals: the PI3Ki LY294002 (1 μM), the JNKi SP600125 (10 μM), the NFκBi BAY 11-7082 (4 μM), and the LCKi RK-24466 (0.5 μM).

### Virus production

HEK293T cells were grown to approximately 70‒80% confluence prior to transfection. Transfection was performed using FuGENE 6 (Promega) and Opti-MEM (Gibco) according to the manufacturer’s recommendations. The helper plasmid pCL-Eco was a gift from Inder Verma (Addgene plasmid # 12371; http://n2t.net/addgene:12371; RRID:Addgene_12371). pLHCX-FLAG-mPKM2 was a gift from Lewis Cantley & Matthew Vander Heiden (Addgene plasmid # 44239; http://n2t.net/addgene:44239; RRID: Addgene_44239). The murine TCR OTI-2A.pMIG II plasmid was a gift from Dario Vignali (Addgene plasmid # 52111; http://n2t.net/addgene:52111; RRID: Addgene_52111). The generation of FLAG-ARS2 was previously described [18], and pBABE-puro was a gift from Hartmut Land, Jay Morgenstern and Bob Weinberg (Addgene plasmid # 1764; http://n2t.net/addgene:1764; RRID: Addgene_1764) and was used as an empty vector control. RT3GEPIR was a gift from Johannes Zuber (Addgene plasmid # 111169; http://n2t.net/addgene:111169; RRID: Addgene_111169) and was modified to express shRNAs as described below. Sixteen hours posttransfection, the medium was replaced, and medium containing viral particles was collected 24‒48 h later.

### Retroviral transduction of CD8+ T cells

To generate OVA-specific OT-I TCR-expressing T cells, CD8^+^ T cells were isolated and activated for 24 h as described above prior to 2 days of transduction with a retroviral vector containing the alpha and beta chains of the OT-I TCR using an established protocol [50]. The transduced cells were expanded for 7 days prior to adoptive transfer into tumor-bearing mice (see below). Rescue experiments were performed following the same transduction protocol to introduce retroviral vectors into CD28^AYAA^ T cells. Three days following the initiation of transduction, T cells were collected for flow cytometric analysis and RNA extraction.

### shRNA-modified cell lines

Custom short hairpin RNAs (shRNAs) targeting ARS2 and CBP80 were designed according to the DSIR algorithm with subsequent filtering with “sensor rules” according to Fellmann et al. [51]. The matched controls had the same sequence except that bases 9–11 were replaced with their complements, as described in Buehler et al. [52]. Oligonucleotides were synthesized by IDT and PCR amplified to generate miR-E shRNA inserts. The PCR products were gel purified and were then cloned and inserted into the retroviral backbone RT3GEPIR by standard cloning techniques. The resulting retroviral vectors were transduced into FL5.12 cells or Bcl-xL-overexpressing FL5.12 variant FL5.12.xL. Stable cell lines were selected using puromycin and isolated by FACS. To induce ARS2 or CBP80 KD, cells stably expressing inducible ARS2, CBP80, or control C9-11 shRNA were treated with 1 µg/mL doxycycline for 72 h.

### siRNA knockdown in human T cells

Healthy donor T cells were purified from peripheral blood mononuclear cells as described above. Up to 10 × 10^6^ purified T cells were transfected in 100 μL aliquots containing a final concentration of 2 μM AllStars Negative Control siRNA (QIAGEN, 1027295) or ARS2 siRNA [12, 18, 20] using a Lonza 4D-Nulecofector and the P3 Primary Cell 4D Nucleofector X Kit (V4XP-3024). Following transfection, the cells were incubated for 5 h in complete T-cell medium, collected by centrifugation, resuspended at 1 × 10^6^ cells/mL in T-cell medium supplemented with T-cell TransAct (Miltenyi 130-111-160) at the manufacturer’s recommended concentration, and collected 72 h later for subsequent experiments.

### qRT‒PCR

RNA was isolated using the miRNeasy Mini Kit (Qiagen), and cDNA was synthesized using SuperScript IV Reverse Transcriptase. Contaminating DNA was removed using RNase-Free DNase (Qiagen), and qPCR was performed using the QuantStudio 6 Flex Real-Time PCR System with SYBR Green (Thermo Fisher Scientific). Target RNA expression was normalized to that of TBP, and relative expression was calculated using the ΔCT or ΔΔCT method. The sequences of the primers used are listed in Table S10.

### RNA immunoprecipitation (RIP)

RIP assays were performed using mouse or human T cells or with FL5.12 or FL5.12.xL cells expressing doxycycline-inducible shRNAs or the C9-11 control shRNA. For ARS2 shRNA-mediated knockdown studies, FL5.12.xL cells were subjected to IL-3 withdrawal for 5 days, after which 0.35 ng/mL IL-3 was added for 72 h. The cells were collected and washed with PBS, and RIP was performed using the RiboCluster Profiler RIP-Assay Kit (MBL) and antibodies specific for ARS2, SRSF3, and hnRNPA1 as well as control IgG (Table S11). Equal amounts of protein were used for each RIP reaction, and aliquots were kept for use as input samples as well as for post-IP QC samples. Equal amounts of input RNA from each sample were used to generate qRT‒PCR standard curves, bound RNAs were quantified by qRT‒PCR, and % input values were calculated based on the standard curves.

### Western blotting

Cells were collected and washed with PBS prior to lysis in RIPA buffer supplemented with Halt^TM^ protease and phosphatase inhibitor (Thermo Fisher). The protein concentrations in the whole-cell extracts were measured by a BCA assay (Thermo Fisher), and equal amounts of protein were separated via SDS‒PAGE. Proteins were transferred to nitrocellulose or PVDF membranes using a Pierce Power Station or standard wet transfer apparatus (Bio-Rad). The membranes were blocked in TBST with 3% milk for 20 min, washed, incubated with the primary antibodies listed in Table S11 overnight at 4 °C, incubated with secondary antibodies, and imaged using a LI-COR Odyssey FC digital imaging system (LI-COR). Quantification was performed using LI-COR imaging software and/or ImageJ software.

### Flow cytometry

Cells were stained with fluorochrome-conjugated antibodies to detect surface-expressed proteins on ice for 20 min prior to washing and resuspension in FACS buffer. For intracellular cytokine staining, cells were stimulated with 50 ng/mL PMA and 500 ng/mL ionomycin (Sigma) for 4 h in the presence of brefeldin A. The cells were surface stained and then fixed/permeabilized using the Biolegend Fixation/Permeabilization Kit according to the manufacturer’s directions for intracellular staining. The antibodies used are listed in Table S11. Data were collected using Becton Dickinson LSRII flow cytometers and analyzed using FlowJo v10.6.1 software.

### RNA sequencing and data analysis

Total RNA was quantified using a Qubit 3.0 fluorometer (Thermo Fisher Scientific), and RNA quality was assessed using TapeStation Analysis Software A.02.02. Sequencing libraries were prepared from 100 ng of total RNA using an RNA HyperPrep Kit with RiboErase (HMR) (Roche Sequencing Solutions). According to the manufacturer’s instructions, rRNA depletion was performed, followed by DNase digestion to remove contaminating gDNA. RNA in the samples was then purified, fragmented, and primed for cDNA synthesis. The fragmented RNA was then reverse transcribed into first-strand cDNA using random primers. Next, the RNA template was replaced, and dUTP was incorporated in place of dTTP to generate double-stranded (ds) cDNA. Pure Beads (KAPA Biosystems) were used to separate the ds cDNA from the second-strand reaction mixture. Multiple indexing adapters were then ligated to the ends of the ds cDNA, preparing them for hybridization onto a flow cell. Adapter-ligated libraries were amplified by PCR, purified using Pure Beads, and validated for the appropriate size with a 4200 TapeStation D1000 Screentape assay (Agilent Technologies, Inc.). The DNA libraries were then quantified using a KAPA Biosystems qPCR kit, pooled in an equimolar ratio, denatured and diluted to 350 pM with the addition of 1% PhiX control library. The pools were loaded into a 200 cycle NovaSeq Reagent cartridge for 100 base pair paired-end sequencing and sequenced in a NovaSeq 6000 instrument following the manufacturer’s recommended protocol (Illumina, Inc.). The resulting data were demultiplexed, and quality control of the raw reads was performed with FastQC (2015). STAR Aligner [53] was used to generate a genome index and BAM files from the paired-end reads using Genome Reference Consortium Build 38 (mm10). Count matrices were generated using RSEM [54]. Normalization and differential gene expression (DEG) analysis were performed in R using the limma package [55, 56]. Genes with an average expression level across all samples exceeding 10 counts per million (CPM) were considered to be expressed and were included in the analyses. Proteins with a > 1 Log_2_-fold change (log2FC) in expression relative to the WT D0 value and an adjusted p value of < 0.05 were considered differentially expressed in activated WT T cells. Changes in pre-mRNA alternative splicing were detected using the percent spliced-in (PSI)-Sigma [24] method. Unique identifiers for splice sites were generated by concatenating event region and target exon parameters. To account for gene expression in splicing analyses, the splicing score for each gene was calculated as PSI × average Log_2_CPM. To determine which changes in gene expression and alternative splicing were shared among activated T cells of different genotypes, Venn diagrams were generated using in-house R scripts and the R package VennDiagram [57]. To allow for some variance in differential gene expression between genotypes, Log_2_FC > 0.847997 (90% of Log2FC = 1) and adj. *p* value <0.05 were set as the cutoff criteria for significance compared to the log2FC values in WT T cells. Similarly, alternative splicing events with PSI values greater than 10 were considered different between genotypes regardless of the p value, while splicing events with PSI values between 5 and 10 were considered different only if the *p* value or the direction of change in the PSI relative to the Day 0 value was different between genotypes.

### [U-^13^C] glucose tracing

CD8^+^ T cells of the indicated genotypes were isolated, activated using αCD3/αCD28-coated microbeads + rIL-2, and cultured for 72 h as described above. On Day 3, the activated T cells were collected, washed in glucose-free RPMI 1640 medium supplemented with 10% dialyzed FBS, 10 U/mL pen/strep, 2 mM L-glutamine, 50 μM β-mercaptoethanol, and 10 mM HEPES (Thermo Fisher Scientific), and resuspended at 3 × 10^6^ cells/mL in the same glucose-free medium supplemented with 2 g/L [U-^13^C] glucose (Cambridge Isotope Laboratories). After 30–120 min, the cells were collected, washed twice in ice-cold PBS and once with ultrapure water and then snap frozen in liquid nitrogen. The frozen pellets were shipped on dry ice to the University of Kentucky Resource Center for Stable Isotope-Resolved Metabolomics, where they were analyzed. Metabolites were extracted from the frozen pellets using a modified Folch method for polar and nonpolar metabolites via the acetonitrile:H_2_O:chloroform (2:1.5:1, v/v) solvent partitioning method as described previously [58, 59]. Via this method, proteins were also obtained as precipitates, which were solubilized with 62.5 mM Tris + 2% sodium dodecyl sulfate (SDS) + 1 mM dithiothreitol (DTT) and denatured at 90 °C for 10 min. The protein concentrations in the extracts were determined using the BCA method (Pierce Chemicals) to calculate the total protein content for normalizing the metabolite content. The polar layer was centrifuged at 15,000 × g, and the supernatants were lyophilized and stored at −80 °C. For IC-UHR-FTMS analysis of anions, the dry pellet was redissolved in 18 MOhm deionized water as described previously [59] using a Dionex ICS-5000 ion chromatography system coupled to a Thermo Orbitrap Fusion Tribrid Fourier transform mass spectrometer with a mass resolving power of 370,000 at 400 m/z. Metabolite peaks were quantified by integration and referenced to internal standards as described previously [60]. The abundances of metabolites and their isotopologues were normalized to the protein mass determined by microBCA as described above.

### Metabolite uptake/secretion

T cells of the indicated genotypes were activated with αCD3/αCD28/rIL-2 for 72 h, collected, and replated in 96-well U-bottom plates (1 × 10^6^ cells in 250 µL of fresh RPMI 1640 medium supplemented with 10% FBS (Corning), 10 U/mL pen/strep, 2 mM L-glutamine, 50 μM β-mercaptoethanol, and 10 mM HEPES (Thermo Fisher)). Following an 8-h incubation, the plate was spun down, and 200 µL of each supernatant was collected and snap frozen in liquid nitrogen. The frozen supernatants were shipped on dry ice to Memorial Sloan Kettering Cancer Center for measurement of glucose uptake and lactate secretion using a YSI biochemistry analyzer (YSI Life Sciences). Total mmol/hour values were measured and normalized to the cell counts determined after the 8-h incubation.

### Seahorse assays

T cells of the indicated genotypes were activated as previously described for 24–72 h prior to use in Seahorse assays. A total of 1.5 × 10^5^ T cells were resuspended in medium for either the Mito Stress Test or Glycolysis Stress Test before analysis on a Seahorse XFe Analyzer (Agilent). To calculate glycolytic or oxidative phosphorylation activity, the background readings were subtracted, and the differences between the pre- and postglucose addition values (glycolysis) and between the pre- and postoligomycin values (OXPHOS) were calculated. The following reagent concentrations were used. Mito Stress Test: 2 µM oligomycin, 1 µM FCCP, and 0.5 µM rotenone/antimycin; Glycolysis Stress Test: 10 mM glucose, 1 µM oligomycin, and 50 mM 2-DG. The fold changes in metabolic parameters were calculated as the differences between the values in WT T cells and those in modified T cells at 24 h.

### PKM restriction digestion

RNA was isolated from human T cells 24 and 72 h post-stimulation, after which cDNA was synthesized using SuperScript IV Reverse Transcriptase. cDNA was amplified as previously described [28] for 22 amplification cycles. The primers used are listed in Table S10. Following amplification, the reactions were split evenly into 4 aliquots and incubated with either no enzyme, NcoI, PstI (NEB), or both for digestion of cDNA. The products were separated on 6% TBE-urea gels (Invitrogen), and the gels were stained with SYBR Gold for 30 min at room temperature in the dark and imaged using a ChemiDoc XRS system (Bio-Rad). Band densities were quantified using Fiji (ImageJ).

### Quantification and statistical analysis

All the statistical analyses were performed using GraphPad Prism. The detailed statistical analyses, values of n, definitions of center and dispersion, tests performed, and determinations of significance can be found in the figure legends.

### Resource availability

Further information and requests for resources and reagents should be directed to and will be fulfilled by the Lead Contact, Scott H. Olejniczak (arxiv://scott.olejniczak@roswellpark.org).

### Supplementary information

Supplemental Figures

Supplemental Tables

Supplemental uncropped blots

## Data Availability

The datasets generated during this study are available at GSE168824. The data used for RNA-seq analyses is available upon reasonable request.

## Code availability

All plasmids and mouse lines generated in this study will be made available upon reasonable request.
