# Supplementary material for: CD8+ T cell metabolic flexibility elicited by CD28-ARS2 axis-driven alternative splicing of PKM supports antitumor immunity
Source: Cell Mol Immunol. 2024 Jan 18;21(3):260–74. doi: 10.1038/s41423-024-01124-2 (PMC10902291; doi:10.1038/s41423-024-01124-2)
Supplement: Supplementary file 1 — Supplemental Figures [file 41423_2024_1124_MOESM1_ESM.docx]

**Supplemental Figure 1.** *Supplement to Figure 1.* **A.** Heat map visualization of mRNAs coding CBCA components CBP80 (*Ncbp1*), CBP20 (*Ncbp2*), and ARS2 (*Srrt*) in C57BL6/J mouse pan T cells stimulated with αCD3/αCD28 + rIL-2 for indicated number of days. **B.** Western blots showing expression of CBCA components in isolated human pan T cells stimulated with αCD3/αCD28 microbeads for indicated number of days. Representative of three healthy human donors. **C.** Expression of mRNAs coding CBCA components in FACS sorted C57BL/6J CD4^+^ or CD8^+^ T cells stimulated with αCD3/αCD28 + rIL-2 for 24 hrs. **D.** ARS2 (*Srrt*) induction in WT mouse T cells stimulated with either αCD3, αCD28, or both ± 1μg/mL Actinomycin D. **E.** Expression of mRNAs coding CBCA components in human CD8 T cells stratified by male and female; data from DICE database (https://dice-database.org). **F.** Expression of mRNAs coding CBCA components in CD8 T cells from OT-1 TCR transgenic mice at indicated timepoints following infection with LmOVA; data from Immgen dataset GSE15907. **G.** Expression of mRNAs coding CBCA components in naïve CD8 T cells from P14 TCR transgenic mice vs. undivided or divided P14 T cells isolated 36 hrs. following adoptive transfer and infection with acute LCMV Arm; data from GSE216731. **H.** Expression of mRNAs coding CBCA components in naïve CD8 T cells from P14 TCR transgenic mice vs. undivided or divided P14 T cells isolated 48 hrs. following adoptive transfer into TRAMPC1-GP tumor bearing mice; data from GSE216731. **I.** Heat map visualization of expression of mRNAs coding CBCA components in FACS sorted naïve (CD45RA^+^CCR7^+^) human CD8 T cells vs. activated (PD1^+^CD45RA^-^CD28^+^CD39^-^) human CD8 T cells FACS sorted from tumor draining lymph nodes (TDLN) of kidney cancer patients vs. stem-like (PD1^+^CD39^-^ CD28^+^) or terminally differentiated (PD1^+^CD39^+^) human CD8 T cells from kidney tumors; data from GSE216731. **J.** Mean fluorescence intensity (MFI) of indicated cytokines determined by intracellular staining of ARS2 knockdown (red bars) vs. control siRNA transfected (gray bars) human CD8 T cells (n = 4 healthy donors). Bars in **C**, **D**, **F**, **G**, **H**, and **J** represent mean ± SD, dots represent biological replicates. Connected points in **J** represent individual healthy human donors. Differences between groups was determined by ANOVA (**C**, **D**, **E**, **F**, **G**, and **H**) or paired t-tests (**J**). n.s. = not significant, *p < 0.05, **p < 0.01, ***p < 0.001, ****p < 0.0001.

**Supplemental Figure 2.** *Supplement to Figure 1.* **A.** Number of splenocytes found in control (black outlined bars) versus ARS2 knockout (red bars) mice following 5 days of tamoxifen treatment followed by two days of rest. **B.** Viability of control (black outlined bars) versus ARS2 knockout (red bars) T cells following stimulation with αCD3/αCD28 + rIL-2, as measured by DAPI exclusion using flow cytometry. **C.** Representative flow histogram showing CD69 expression on the surface of control (ARS2^f/f^) versus ARS2 knockout T cells 24 hours post-activation (left) and quantification of CD44 surface expression 72 hours post-activation of control (black outlined bars) versus ARS2 knockout (red bars) T cells (right). **D.** Quantification of cell size in femtoliters (fL) over 5 days following activation of control (black line) versus ARS2 knockout (red line) T cells. Connected points represent mean ± SD of 3 biological replicates. **E.** Example gating strategy used to quantify CD8 T cell differentiation status and cytokine expression. **F.** Flow cytometry assessment of naïve (T_N_ = CD62L^hi^, CD44^-^) and effector (TE = CD62L^low^, CD44^+^) phenotype CD8 T cells 72 hours following αCD3/αCD28 + rIL-2 stimulation of isolated ARS2^fl/fl^ or ARS2^KO^ T cells. **G.** Frequency (left) and intensity (right) of IFNγ and IL-2 expression in *ex vivo* activated ARS2^fl/fl^ or ARS2^KO^ T_E_ cells (CD8^+^CD44^+^CD62L^-^). **H.** Frequency (left) of IFNγ^+^TNFα^+^ 3 days following *ex vivo* activation of CD8 T cells isolated from tamoxifen treated ARS2^f/f^, CreERT2^+^ mT/mG Cre reporter mice. Mean fluorescence intensity (MFI) of IFNγ expression in IFNγ^+^CD8^+^ T cells (middle) or of TNFα expression in TNFα^+^CD8^+^ T cells. Cre^-^ (mT) T cells (red bars) were defined by gating on mT^+^mG^-^CD8^+^ T cells and represent those lacking Cre activity and therefore expressing wild-type levels of ARS2 while Cre^+^ (mG) T cells (green bars) were identified by gating on mG^+^CD8^+^ T cells and represent those where Cre activity led to ARS2 deletion. **I.** Representative flow histogram showing co-expression of GFP transduction marker in OT-I transduced ARS2^f/f^ or ARS2^KO^ CD8^+^ T cells used in **Fig. 1I**. Bars in **A**, **B,** **C**, **F**, **G**, and **H** represent mean ± SD, dots represent biological replicates. Connected points in **H** represent individual mice. Differences between groups was determined by ANOVA (**B**, **F**, and **G**) or t-tests (**A** and **C**, unpaired; **H**, paired). n.s. = not significant, *p < 0.05, **p < 0.01, ***p < 0.001, ****p < 0.0001.

**Supplemental Figure 3.** *Supplement to Figure 2.* **A.** Expression of ARS2 mRNA (*Srrt*) in freshly isolated WT and CD28 mutant mouse T cells relative to endogenous control (*Tbp*). **B.** Change in ARS2 mRNA (*Srrt*) expression in CD28 knockout T cells stimulated with either αCD3/αCD28 + rIL-2 or PMA/Ionomycin for 24 hours. **C.** Representative flow histogram showing co-expression of GFP transduction marker in OT-I transduced WT, CD28^AYAA^ knockin or CD28^Y170F^ knockin CD8^+^ T cells used in **Fig. 2F**. **D.** Growth of EL4 tumors implanted on the flank opposite E.G7-OVA tumors shown in **Fig. 2F**. **E.** Relative IFNγ (left) and IL-2 (right) protein and mRNA expression in WT and CD28 mutant T cells on day 3 of activation. Bars in **A**, **B**, and **E** represent mean ± SD, dots represent biological replicates. Lines in **D** represent individual tumors. Differences between groups was determined by ANOVA (**A** and **E**), mixed-effects analysis (**D**), or student’s t test (**B**). n.s. = not significant, **p < 0.01, ****p < 0.0001.

**Supplemental Figure 4.** *Supplement to Figure 3.* **A.** Frequency of T cell activation-induced alternative splicing (AS) events by type of alternative splice - as determined by PSI-Sigma analysis of RNA-seq data - that occur in an ARS2-dependent (red bars) or ARS2-independent (black bars) manner. **B.** Venn diagrams showing co-regulation of AS events by ARS2 (red), CD28-PYAP intracellular domain signaling (green), and CD28-YMNM intracellular domain signaling (blue) on day 1 (left) or day 3 (right) of activation. **C.** Bar chart showing quantification of **B** plus CD28 independent alternative splicing events. **D.** RT-PCR amplification followed by restriction digest with NcoI (N), PstI (P), or both (NP) was used to quantify *PKM1* and *PKM2* mRNA in human T cells at day 1 or day 3 post activation. Ratio of *PKM2/PKM1* is shown below as mean ± SD of 3 independent healthy donors. **E.** Total *Pkm* mRNA expression determined by qRT-PCR analysis of day 3 samples shown in **Fig. 3D**. **F.** RNA binding sites identified by crosslinking immunoprecipitation and sequencing for ARS2 (GSE94427), SRSF3 (GSE118265), hnRNPA1 (ENCSR154HRN), and PTBP1 (ENCSR981WKN) in HEK 293T cells mapped to exons 8 through 11 of the human *PKM* transcript. **G.** RNA immunoprecipitation (RIP) of RNP complexes from FL5.12 cells using antibodies to indicated proteins, control rabbit IgG (rIgG), or non-targeting Sp2/0 mouse monoclonal antibody followed by qRT-PCR to detect *Pkm1* or *Pkm2* pre-mRNA. Primers used span intron-exon boundaries: pre-*Pkm1* = intron 8 exon 9, pre-*Pkm2* = intron 9 to exon 10 (see **Table S10**). **H.** ARS2 RIP from day 3 activated mouse T cells followed by qRT-PCR using pre-*Pkm2* primers. **I.** ARS2 RIP-qRT-PCR as in **H** using human T cells and human pre-*PKM* primers. **J.** ARS2 RIP from CBP80 shRNA knockdown vs. control shRNA expressing FL5.12 cells followed by qRT-PCR using pre-*Pkm2* primers. **K.** SRSF3 and hnRNPA1 RIPs from ARS2 knockdown vs. control shRNA expressing FL5.12.xL cells stimulated with IL-3 for three days followed by qRT-PCR using pre-*Pkm2* primers. Bars in **E**, **G**, **H**, **I**, **J**, and **K** represent mean ± SD, **G**, **H**, **I**, **J**, and **K** show representative RIPs repeated at least 2 times, dots in **E** represent biological replicates. Differences between groups was determined by ANOVA (**E**, **J**, and **K**). n.s. = not significant, *p < 0.05, ***p < 0.001, ****p < 0.0001.

**Supplemental Figure 5.** *Supplement to Figure 4.* **A.** Validation of PKM2 inducible knockout model showing reduced expression of *Pkm2* (left) and increased expression of *Pkm1* (right) in T cells purified from Pkm2-floxed (Pkm2^f/f^) versus Pkm2-floxed mice expressing CreERT2 (Pkm2^KO^) following 5 days of tamoxifen (100 μg/day *i.p*.) treatment and 2 days of rest. **B.** PKM1 and PKM2 protein expression in T cells as in **A**. **C.** Number of splenocytes in mice as in **A**. **D.** Frequency of peripheral T cells in spleens of mice as in **A**. **E.** T cell activation marker expression in PKM2^KO^ vs. floxed control T cells stimulated for 24 hours (CD69, left) or 72 hours (CD44, right) using αCD3/αCD28 coated beads + rIL-2 **F.** IFNγ and IL-2 mRNA (white bars) and protein (gray bars) expression in day 3 activated PKM2^KO^ T cells relative to floxed control T cells. **G.** Growth of EL4 tumors implanted on the flank opposite E.G7-OVA tumors shown in **Fig. 4B**. **H.** Overexpression of *Pkm2* as determined by qRT-PCR in mouse T cells of indicated genotypes 3 days following activation and transduction with pLHCX-FLAG-mPKM2. **I, J, K.** Increased ^13^C-glucose labeling in isotopomers of pentose phosphate pathway metabolites upstream of PKM2 in day 3 activated PKM2^KO^ CD8 T cells. **L.** Lack of ^13^C-glucose labeling of glutathione in day 3 activated CD8 T cells. Bars in **A**, **C**, **D**, **E**, **F**, **H**, **I**, **J**, **K**, and **L** represent mean ± SD, **A** shows representative qRT-PCR results repeated at least 2 times, dots in **C**, **D**, **E**, **F**, **I**, **J**, **K**, and **L** represent biological replicates. Lines in **G** represent individual tumors. Differences between groups was determined by ANOVA (**F**, **G**, **H**, **I**, **J**, **K**, and **L**) or student’s t test (**A**, **C**, **D**, and **E**). n.s. = not significant, *p < 0.05, **p < 0.01, ***p < 0.001, ****p < 0.0001.

**Supplemental Figure 6.** *Supplement to Figure 5.* **A.** Ratio of *Pkm2* to *Pkm1* mRNA expression determined by qRT-PCR in T cells freshly isolated (Day 0) from mice of indicated genotype or T cells activated with αCD3/αCD28 coated beads + rIL-2 for 3 days (Day 3). **B.** Lack of ^13^C-glucose labeling of glutathione in day 3 activated CD8 T cells. **C, D, E.** No change in ^13^C-glucose labeling in isotopomers of pentose phosphate pathway metabolites upstream of PKM2 in day 3 activated ARS2^KO^ or CD28^AYAA^ CD8 T cells.
