# Supplementary material for: CD8+ T cell metabolic flexibility elicited by CD28-ARS2 axis-driven alternative splicing of PKM supports antitumor immunity
Source: Cell Mol Immunol. 2024 Jan 18;21(3):260–74. doi: 10.1038/s41423-024-01124-2 (PMC10902291; doi:10.1038/s41423-024-01124-2)
Supplement: Supplementary file 3 — Supplemental uncropped blots [file 41423_2024_1124_MOESM3_ESM.docx]

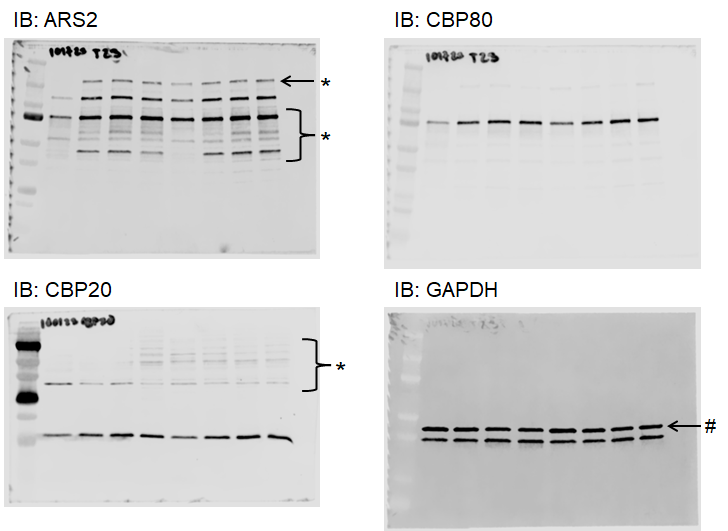


**Uncropped Western blots from Figure 1B. Lane 1:** protein ladder, **Lane 2:** resting (d0) human CD4^+^ T cells, **Lane 3:** d1 activated human CD4^+^ T cells, **Lane4:** d2 activated human CD4^+^ T cells, **Lane 5:** d3 activated human CD4^+^ T cells. **Lane 6:** resting (d0) human CD8^+^ T cells, **Lane 7:** d1 activated human CD8^+^ T cells, **Lane8:** d2 activated human CD8^+^ T cells, **Lane 9:** d3 activated human CD8^+^ T cells. *non-specific bands, # remaining signal from re-probe of β-Actin blot.


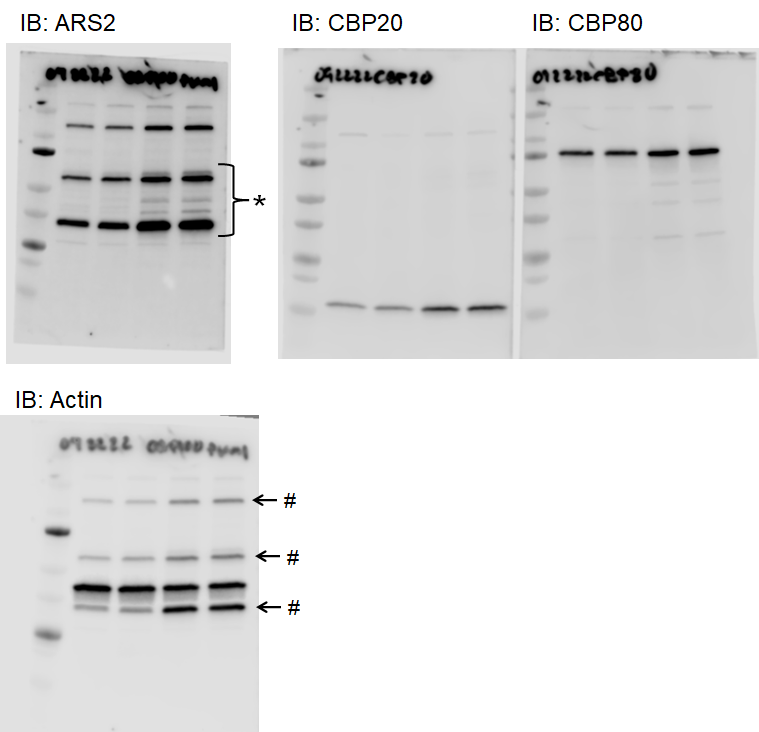


**Uncropped Western blots from Supplemental Figure 1B. Lane 1:** protein ladder, **Lane 2:** resting (d0) T cells, **Lane 3:** d1 activated T cells, **Lane4:** d2 activated T cells, **Lane 5:** d3 activated T cells. *non-specific bands, # remaining signal from re-probe of ARS2 blot.


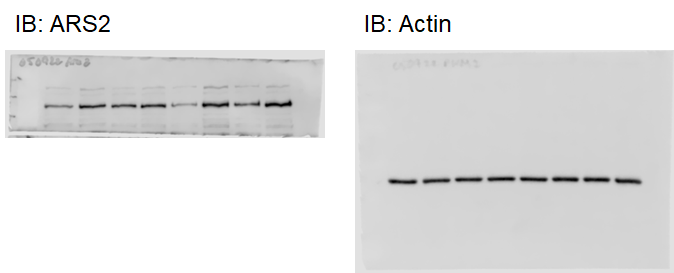


**Uncropped Western blots from Figure 2E.** **Lane 1:** protein ladder, **Lane 2:** d1 activated ARS2^iKO^ mouse T cells, **Lane 3:** d1 activated ARS2^fl/fl^ mouse T cells, **Lane 4:** d1 activated CD28^AYAA^ mouse T cells, **Lane 5:** d1 activated CD28^Y170F^ mouse T cells, **Lane 6:** d3 activated ARS2^iKO^ mouse T cells, **Lane 7:** d3 activated ARS2^fl/fl^ mouse T cells, **Lane 8:** d3 activated CD28^AYAA^ mouse T cells, **Lane 9:** d3 activated CD28^Y170F^ mouse T cells. **Membrane for ARS2 blot was cut at ~80 kDa to facilitate simultaneous probing of large (ARS2) and small proteins.


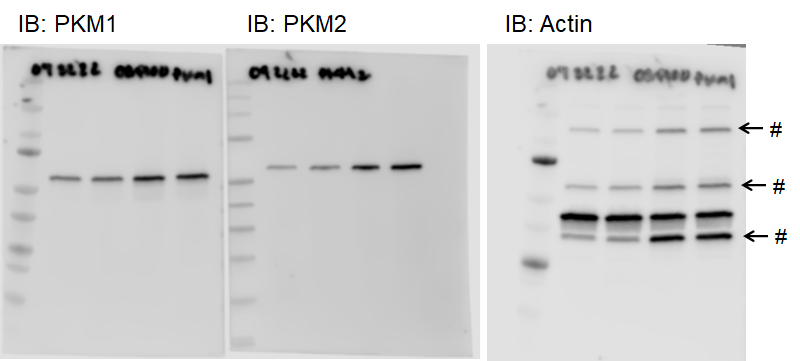


**Uncropped Western blots from Figure 3C***.* **Lane 1:** protein ladder, **Lane 2:** resting (d0) human T cells, **Lane 3:** d1 activated human T cells, **Lane4:** d2 activated human T cells, **Lane 5:** d3 activated human T cells. # remaining signal from re-probe of ARS2 blot.


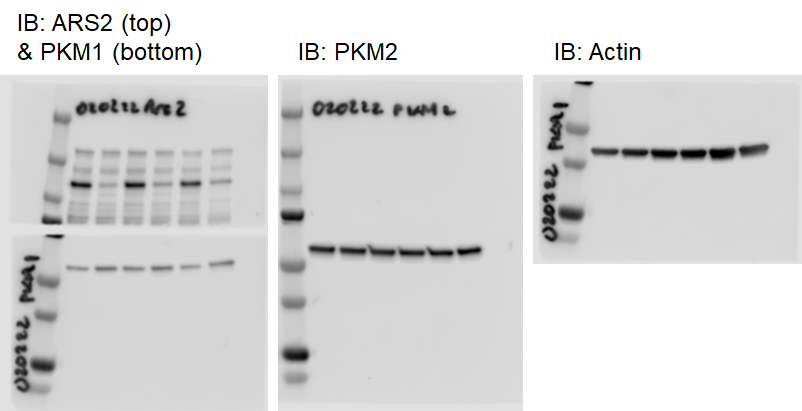


**Uncropped Western blots from Figure 3G.** **Lane 1:** protein ladder, **Lane 2:** d3 activated control siRNA transfected human T cells, **Lane 3:** d3 activated ARS2 siRNA transfected human T cells, **Lane4:** d4 activated control siRNA transfected human T cells, **Lane 5:** d4 activated ARS2 siRNA transfected human T cells, **Lane 6:** d5 activated control siRNA transfected human T cells, **Lane 7:** d5 activated ARS2 siRNA transfected human T cells.


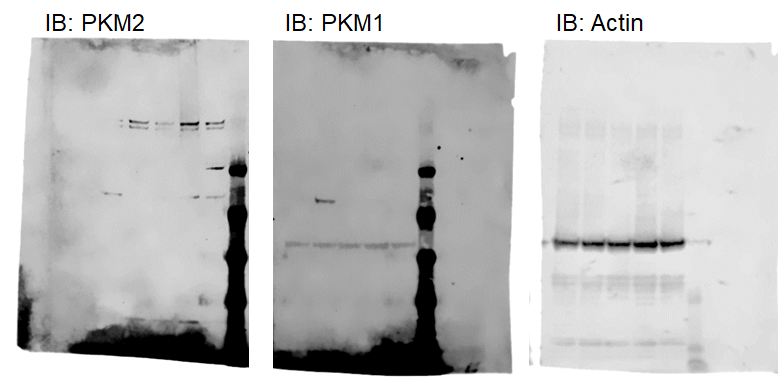


**Uncropped Western blots from Supplemental Figure 5B. Lane 1:** Pkm2^fl/fl^ T cells, **Lane 2:** Pkm2^KO^ T cells, **Lanes 3 – 5:** Unrelated samples, **Lane 6:** protein ladder.


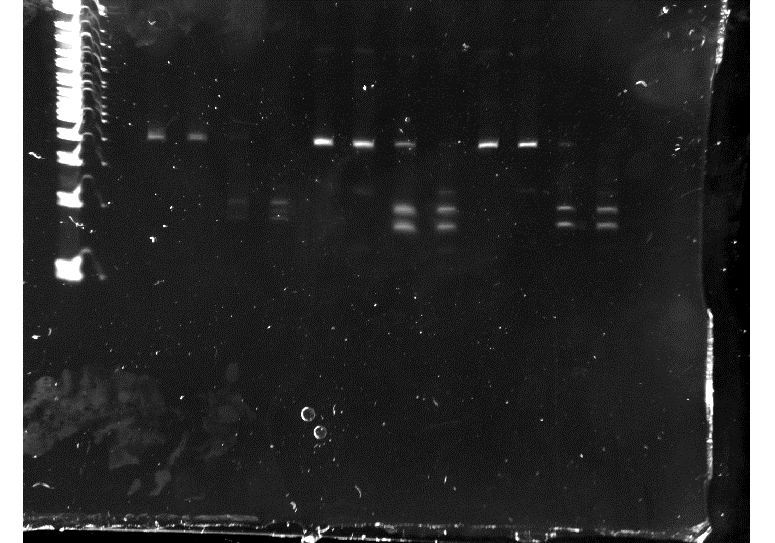


**Uncropped EtBr-stained 2% agarose DNA gel photo from Supplemental Figure 3D.** **Lane 1:** DNA ladder, **Lane 2:** no sample, **Lane 3:** undigested *PKM* cDNA from resting (d0) human T cells, **Lane 4:** NcoI digested *PKM* cDNA from resting (d0) human T cells, **Lane 5:** PstI digested *PKM* cDNA from resting (d0) human T cells, **Lane 6:** NcoI + PstI digested *PKM* cDNA from resting (d0) human T cells, **Lane 7:** undigested *PKM* cDNA from d1 activated human T cells, **Lane 8:** NcoI digested *PKM* cDNA from d1 activated human T cells, **Lane 9:** PstI digested *PKM* cDNA from d1 activated human T cells, **Lane 10:** NcoI + PstI digested *PKM* cDNA from d1 activated human T cells, **Lane 11:** undigested *PKM* cDNA from d3 activated human T cells, **Lane 12:** NcoI digested *PKM* cDNA from d3 activated human T cells, **Lane 13:** PstI digested *PKM* cDNA from d3 activated human T cells, **Lane 14:** NcoI + PstI digested *PKM* cDNA from d3 activated human T cells.
